# Surveillance of Vaccination Coverage among Adult Populations — United States, 2015

**DOI:** 10.15585/mmwr.ss6611a1

**Published:** 2017-05-05

**Authors:** Walter W. Williams, Peng-Jun Lu, Alissa O’Halloran, David K. Kim, Lisa A. Grohskopf, Tamara Pilishvili, Tami H. Skoff, Noele P. Nelson, Rafael Harpaz, Lauri E. Markowitz, Alfonso Rodriguez-Lainz, Amy Parker Fiebelkorn

**Affiliations:** 1 Immunization Services Division, National Center for Immunization and Respiratory Diseases, CDC; 2 Leidos, Inc, Atlanta, GA; 3 Influenza Division, National Center for Immunization and Respiratory Diseases, CDC; 4 Division of Bacterial Diseases, National Center for Immunization and Respiratory Diseases, CDC; 5 Division of Viral Hepatitis, National Center for HIV/AIDS, Viral Hepatitis, STD, and TB Prevention, CDC; 6 Division of Viral Diseases, National Center for Immunization and Respiratory Diseases, CDC; 7 Division of Global Migration and Quarantine, National Center for Emerging and Zoonotic Infectious Diseases, CDC

## Abstract

**Problem/Condition:**

Overall, the prevalence of illness attributable to vaccine-preventable diseases is greater among adults than among children. Adults are recommended to receive vaccinations based on their age, underlying medical conditions, lifestyle, prior vaccinations, and other considerations. Updated vaccination recommendations from CDC are published annually in the U.S. Adult Immunization Schedule. Despite longstanding recommendations for use of many vaccines, vaccination coverage among U.S. adults is low.

**Period Covered:**

August 2014–June 2015 (for influenza vaccination) and January–December 2015 (for pneumococcal, tetanus and diphtheria [Td] and tetanus and diphtheria with acellular pertussis [Tdap], hepatitis A, hepatitis B, herpes zoster, and human papillomavirus [HPV] vaccination).

**Description of System:**

The National Health Interview Survey (NHIS) is a continuous, cross-sectional national household survey of the noninstitutionalized U.S. civilian population. In-person interviews are conducted throughout the year in a probability sample of households, and NHIS data are compiled and released annually. The survey objective is to monitor the health of the U.S. population and provide estimates of health indicators, health care use and access, and health-related behaviors.

**Results:**

Compared with data from the 2014 NHIS, increases in vaccination coverage occurred for influenza vaccine among adults aged ≥19 years (a 1.6 percentage point increase compared with the 2013–14 season to 44.8%), pneumococcal vaccine among adults aged 19–64 years at increased risk for pneumococcal disease (a 2.8 percentage point increase to 23.0%), Tdap vaccine among adults aged ≥19 years and adults aged 19–64 years (a 3.1 percentage point and 3.3 percentage point increase to 23.1% and to 24.7%, respectively), herpes zoster vaccine among adults aged ≥60 years and adults aged ≥65 years (a 2.7 percentage point and 3.2 percentage point increase to 30.6% and to 34.2%, respectively), and hepatitis B vaccine among health care personnel (HCP) aged ≥19 years (a 4.1 percentage point increase to 64.7%). Herpes zoster vaccination coverage in 2015 met the *Healthy People 2020* target of 30%. Aside from these modest improvements, vaccination coverage among adults in 2015 was similar to estimates from 2014. Racial/ethnic differences in coverage persisted for all seven vaccines, with higher coverage generally for whites compared with most other groups. Adults without health insurance reported receipt of influenza vaccine (all age groups), pneumococcal vaccine (adults aged 19–64 years at increased risk), Td vaccine (adults aged ≥19 years, 19–64 years, and 50–64 years), Tdap vaccine (adults aged ≥19 years and 19–64 years), hepatitis A vaccine (adults aged ≥19 years overall and among travelers), hepatitis B vaccine (adults aged ≥19 years, 19–49 years, and among travelers), herpes zoster vaccine (adults aged ≥60 years), and HPV vaccine (males and females aged 19–26 years) less often than those with health insurance. Adults who reported having a usual place for health care generally reported receipt of recommended vaccinations more often than those who did not have such a place, regardless of whether they had health insurance. Vaccination coverage was higher among adults reporting one or more physician contacts in the past year compared with those who had not visited a physician in the past year, regardless of whether they had health insurance. Even among adults who had health insurance and ≥10 physician contacts within the past year, depending on the vaccine, 18.2%–85.6% reported not having received vaccinations that were recommended either for all persons or for those with specific indications. Overall, vaccination coverage among U.S.-born adults was higher than that among foreign-born adults, with few exceptions (influenza vaccination [adults aged 19–49 years and 50–64 years], hepatitis A vaccination [adults aged ≥19 years], and hepatitis B vaccination [adults aged ≥19 years with diabetes or chronic liver conditions]).

**Interpretation:**

Coverage for all vaccines for adults remained low but modest gains occurred in vaccination coverage for influenza (adults aged ≥19 years), pneumococcal (adults aged 19–64 years with increased risk), Tdap (adults aged ≥19 years and adults aged 19–64 years), herpes zoster (adults aged ≥60 years and ≥65 years), and hepatitis B (HCP aged ≥19 years); coverage for other vaccines and groups with vaccination indications did not improve. The 30% *Healthy People 2020* target for herpes zoster vaccination was met. Racial/ethnic disparities persisted for routinely recommended adult vaccines. Missed opportunities to vaccinate remained. Although having health insurance coverage and a usual place for health care were associated with higher vaccination coverage, these factors alone were not associated with optimal adult vaccination coverage. HPV vaccination coverage for males and females has increased since CDC recommended vaccination to prevent cancers caused by HPV, but many adolescents and young adults remained unvaccinated.

**Public Health Actions:**

Assessing factors associated with low coverage rates and disparities in vaccination is important for implementing strategies to improve vaccination coverage. Evidence-based practices that have been demonstrated to improve vaccination coverage should be used. These practices include assessment of patients’ vaccination indications by health care providers and routine recommendation and offer of needed vaccines to adults, implementation of reminder-recall systems, use of standing-order programs for vaccination, and assessment of practice-level vaccination rates with feedback to staff members. For vaccination coverage to be improved among those who reported lower coverage rates of recommended adult vaccines, efforts also are needed to identify adults who do not have a regular provider or insurance and who report fewer health care visits.

## Introduction

Overall, the prevalence of illness attributable to vaccine-preventable diseases is greater among adults aged ≥19 years than among children aged ≤12 years (*1*–*5*) attributable in great part to successful childhood vaccination programs. The prevalence of vaccine-preventable illnesses among older persons is especially high (*1*–*4*). Vaccinations are recommended throughout a person’s lifetime to prevent vaccine-preventable diseases and their sequelae. However, adult vaccination coverage remains low for most routinely recommended vaccines (*5*) and below *Healthy People 2020* targets (https://www.healthypeople.gov/2020/topics-objectives/topic/immunization-and-infectious-diseases). In October 2016, the Advisory Committee on Immunization Practices (ACIP) approved the adult immunization schedule for 2017 (*6*). Influenza vaccination is recommended for all adults each year. Other adult vaccinations are recommended for specific populations based on a person’s age, health conditions, behavioral risk factors (e.g., injection drug use), occupation, travel, and other indications (*6*).

In February 2016, CDC released the first comprehensive report of adult vaccination coverage that described associations related to respondents’ characteristics (e.g., demographic and access to care) (*5*). This surveillance summary updates those vaccination coverage estimates. The estimates provided in this report can be used by public health practitioners, adult vaccination providers, and the general public to understand better the factors that contribute to low vaccination rates and modify strategies and interventions to improve vaccination coverage.

## Methods

To assess vaccination coverage among adults aged ≥19 years for selected vaccines and factors associated with vaccination, CDC analyzed data from the 2015 National Health Interview Survey (NHIS); for influenza coverage, data from the 2014 NHIS (for August–December) also were used for the 2014 component of the 2014–15 influenza season. This report highlights the results of that analysis for influenza, pneumococcal, tetanus toxoid-containing (tetanus and diphtheria vaccine [Td] or tetanus and diphtheria with acellular pertussis vaccine [Tdap]), hepatitis A, hepatitis B, herpes zoster (shingles), and human papillomavirus (HPV). Data are reported by selected demographic and access-to-care characteristics (e.g., age, race/ethnicity, indication for vaccination, health insurance status, contacts with physicians, nativity, and citizenship). Other estimates of influenza vaccination coverage using data from 2015–16 and earlier seasons from other sources have been published (*7*–*9*). These data sources have been described previously (*10*). Proportions were estimated of adults aged ≥19 years who received selected vaccinations during 2010–2015. Estimates of proportions vaccinated were stratified by age group, risk status, health insurance status, having a usual place for health care, number of physician contacts, nativity, number of years living in the United States, and citizenship. 

### Data Source and Collection

NHIS collects information about the health and health care of the noninstitutionalized U.S. civilian population using nationally representative samples. Face-to-face interviews are conducted by the U.S. Census Bureau for CDC’s National Center for Health Statistics. The total adult sample was 33,348 persons aged ≥19 years. Additional information on NHIS methods is available at https://www.cdc.gov/nchs/nhis/methods.htm.

Questions about receipt of vaccinations recommended for adults are asked of one randomly selected adult within each family in the household and have been described previously (*5*). A summary is provided of questions asked to ascertain whether adults received influenza, pneumococcal, Td, Tdap, hepatitis A, hepatitis B, herpes zoster (shingles), and human papillomavirus (HPV) vaccines as well as to ascertain classification as health care personnel (HCP), whether respondents had health insurance coverage, and whether there is a place to which respondents usually go when sick or need advice on their health (Appendix). There were no questions in the 2015 NHIS to ascertain pneumococcal vaccination by type of vaccine (23-valent pneumococcal polysaccharide vaccine or 13-valent pneumococcal conjugate vaccine). The presence of selected conditions that increase risk for pneumococcal disease and are defined by ACIP as indications for pneumococcal vaccines (Box 1) (*6*) was determined by responses to questions in NHIS. For hepatitis A and hepatitis B vaccination, data were collected also on selected respondent characteristics that increase the risk for infection (travel to countries in which hepatitis A infections are endemic and having chronic liver disease, travel to countries in which hepatitis B infections are endemic, and having diabetes or chronic liver disease, respectively).

BOX 1 **Selected conditions that increase the risk for pneumococcal disease and are defined as indications for pneumococcal vaccines by the Advisory Committee on Immunization Practices**

Adults were considered at increased risk for pneumococcal disease or its complications if they

- had ever been told by a doctor or other health professional that they had diabetes, emphysema, chronic obstructive pulmonary disease, coronary heart disease, angina, heart attack, or other heart condition;
- had a diagnosis of cancer during the previous 12 months (excluding nonmelanoma skin cancer);
- had ever been told by a doctor or other health professional that they had lymphoma, leukemia, or blood cancer;
- had been told by a doctor or other health professional that they had chronic bronchitis or weak or failing kidneys during the preceding 12 months;
- had an asthma episode or attack during the preceding 12 months; or
- were current smokers.

**Source:** CDC. Advisory Committee on Immunization Practices recommended immunization schedule for adults aged 19 years and older—United States, 2017. MMWR Morb Mortal Wkly Rep 2017;66:1–4.

Vaccination status and demographic and other characteristics (e.g., health conditions, insurance status, and usual source and frequency of health care) are self-reported. Race/ethnicity was categorized as Hispanic or Latino, black, white, Asian, and “other.” Persons identified as Hispanic or Latino might be of any race. Persons identified as black, white, Asian, or other race are non-Hispanic. “Other” includes American Indians/Alaska Natives and persons of multiple race. The five racial/ethnic categories are mutually exclusive. Nativity was categorized as U.S.-born (persons born in one of the 50 states or the District of Columbia) or foreign-born (persons who were not born in the United States).

### Analysis

For the noninfluenza adult vaccination coverage estimates, the weighted proportion of respondents who reported receiving selected vaccinations was calculated. To better assess influenza vaccination coverage for the 2014–15 influenza season, CDC restricted reported coverage to persons who were interviewed during August 2014–June 2015 and vaccinated during July 2014–May 2015, using the Kaplan-Meier survival analysis procedure; 2014 NHIS data for August–December 2014 were used for the 2014 component of the 2014–15 influenza season. Differences were measured as the simple difference between the 2013–14 and 2014–15 influenza seasons. Data for missing months and years of vaccination (3.5%) were imputed.

To assess adjusted vaccination coverage and adjusted prevalence ratios for each vaccine, logistic regression and predicted marginal modeling were used for selected comparisons (health insurance status). Estimates were adjusted for age, sex, race/ethnicity, marital status, education, employment status, poverty level, number of physician contacts in the past year, usual source of health care, self-reported health status, nativity, and region of residence. Income-to-poverty ratio variables are included in the NHIS public use data file (https://www.cdc.gov/nchs/nhis/nhis_2015_data_release.htm). Poverty thresholds were defined according to family size using weighted average census poverty thresholds from 2013, the average consumer price index from 2013, actual consumer price index values for January–July 2014, and projected consumer price index values for August–December 2014 (ftp://ftp.cdc.gov/pub/Health_Statistics/NCHS/Dataset_Documentation/NHIS/2015/srvydesc.pdf).

Weighted data were used to produce national vaccination coverage estimates. Point estimates and 95% confidence intervals (CIs) were calculated by using statistical software to account for the complex sample design, and t tests were used for comparisons between 2015 and 2014 and for comparisons of each level of each characteristic (e.g., race/ethnicity, age group, HCP status, patient care status, access-to-care factors, nativity, years of residence in the United States, and citizenship status) to a chosen referent level (e.g., for race/ethnicity, non-Hispanic white was the reference group). For influenza vaccination, tests for linear trend were performed using a weighted linear regression on the season-specific estimates, using season number as the independent variable and the inverse of the estimated variance of the estimated vaccination coverage as the weights. For vaccination with the other vaccines, tests for linear trend were performed in SUDAAN using the RATIO procedure. Statistical significance was defined as p<0.05. Coverage estimates are not reported for small sample size (n<30) or relative standard error (standard error/estimates) >0.3.

## Results

The final sample adult component response rate for the 2015 NHIS was 55.2%. The final sample adult component response rates for estimating influenza vaccination coverage for the 2014–15 influenza season were 58.9% for 2014 and 55.2% for 2015. The total adult sample for influenza coverage estimation was 31,897 persons aged ≥19 years. Detailed information for vaccination coverage estimates stratified by selected variables is summarized (Box 2). These selected variables include health insurance status, having a usual place for health care, number of physician contacts, age group, nativity, number of years living in the United States, and citizenship.

BOX 2 **Estimated proportion of adults aged ≥19 years who received selected vaccinations, by age group, risk status, health insurance status, having a usual place for health care, physician contacts, nativity, number of years living in the United States, and citizenship — National Health Interview Survey, United States, 2015**

**Table:** 

Supplementary material	Result summary
Association of health insurance status with vaccination coverage among adult populations	Overall, vaccination coverage was generally lower among adults without health insurance compared with those with health insurance. Adult vaccination coverage differed by the type of health insurance. Vaccination coverage was generally higher among adults with private health insurance compared with those reporting public health insurance. Detailed information from these analyses are available at https://stacks.cdc.gov/view/cdc/45000.
Association of health insurance status and having a usual place for health care with vaccination coverage	Generally, adults with a usual place for health care reported having received recommended vaccinations more often than those who did not have a usual place for health care, regardless of whether they had health insurance. Among adults with health insurance, coverage was higher among those who reported having a usual place for health care compared with those who did not have a usual place for health care. Detailed information from these analyses are available at https://stacks.cdc.gov/view/cdc/44999.
Adult vaccination coverage by health insurance status and physician contacts	With a few exceptions (overall hepatitis A vaccination among adults aged ≥19 years and human papillomavirus vaccination among women aged 19–26 years), vaccination coverage was higher among those reporting having had one or more physician contacts in the past year compared with those who had not visited a physician in the past year, regardless of whether they had health insurance. In addition, vaccination coverage generally increased as the number of physician contacts increased. Among adults who had health insurance and ≥10 physician contacts within the past year, 18.2%–85.6% reported not having received vaccinations that were recommended either for all persons or for those with some specific indication. Detailed information from these analyses are available at https://stacks.cdc.gov/view/cdc/44998.
Association of respondent age with adult vaccination coverage	Influenza and pneumococcal vaccination coverage among adults aged ≥65 years was higher compared with coverage among adults aged 19–64 years; however, Td and Tdap coverage among adults aged ≥65 years was lower compared with coverage among adults aged <65 years. Hepatitis B vaccination coverage among adults with diabetes aged ≥60 years was lower compared with coverage among adults aged 19–59 years with diabetes. Herpes zoster coverage among adults aged ≥65 years was higher compared with coverage among adults aged 60–64 years. Detailed information from these analyses are available at https://stacks.cdc.gov/view/cdc/45000, https://stacks.cdc.gov/view/cdc/44999, and https://stacks.cdc.gov/view/cdc/44998.
Adult vaccination coverage adjusted for selected demographic and access-to-care characteristics	Adults without health insurance were less likely than those with health insurance to be vaccinated after adjusting for confounders for influenza (aged ≥19 years); Td (aged ≥19 years); hepatitis A (aged ≥19 years); and hepatitis B (aged ≥19 years). Detailed information from these analyses are available at https://stacks.cdc.gov/view/cdc/44997.
Adult vaccination coverage by nativity, years living in the United States, and citizenship	Overall, vaccination coverage among U.S.-born respondents was higher than that of foreign-born respondents with few exceptions (influenza vaccination [adults aged 19–49 years and aged 50–64 years], hepatitis A vaccination [adults aged ≥19 years], and hepatitis B vaccination [adults aged ≥19 years with diabetes or chronic liver conditions]). Compared with U.S.-born adults, there were large gaps in vaccination coverage among foreign-born adults for HPV vaccination (females aged 19–26 years [44.3% versus 22.8%]) and Tdap vaccination (adults aged ≥ 19 years [25.6% versus 13.3%]). Vaccination status varied by time living in the United States. Coverage among foreign-born adults who were U.S. citizens was generally higher than that for foreign-born respondents who were not U.S. citizens. Detailed information from these analyses are available at https://stacks.cdc.gov/view/cdc/44996.

### Influenza Vaccination Coverage

Influenza vaccination coverage for the 2014–15 season among adults aged ≥19 years was 44.8%, an increase of 1.6 percentage points from the 2013–14 season (Table 1). Coverage among whites aged ≥19 years was higher (48.5%) than that for blacks (37.7%) and Hispanics (33.0%). Influenza coverage was 32.5% among adults aged 19–49 years and 48.7% among adults aged 50–64 years. Coverage among adults aged ≥65 years (73.5%) was higher compared with younger age groups. Among HCP aged ≥19 years, influenza vaccination coverage overall was 68.6%, similar to the estimate for the 2013–14 season (Table 2). Among HCP aged ≥19 years with and without direct patient care responsibilities, influenza vaccination coverage was 68.9% and 67.9%, respectively, similar to 2013–14 estimates (Table 3). Influenza coverage among white HCP with direct patient care was higher (72.8%) than that for black (53.8%) and Hispanic (59.8%) HCP with direct patient care. Coverage was 72.0% for black HCP aged ≥19 years without direct patient care responsibilities, a 28.1 percentage point increase compared with the 2013–14 estimate. However, for the 2014–15 season, influenza vaccination coverage among HCP without direct patient care responsibilities was similar across all racial/ethnic groups (Table 3). During the 2009–10 through the 2014–15 influenza seasons, fewer than half of adults aged ≥19 years were vaccinated (range: 37.2%–44.8%). Among HCP, 56.6%–68.6% reported influenza vaccination during this period. Linear trend tests indicated influenza vaccination coverage among persons aged ≥19 years and HCP aged ≥19 years increased from the 2009–10 to 2014–15 influenza seasons (p<0.01 and p = 0.04, respectively) (see https://stacks.cdc.gov/view/cdc/44995).

**TABLE 1:** Estimated proportion of adults aged ≥19 years who received selected vaccinations, by age group, increased-risk status,* and race/ethnicity^†^ — National Health Interview Survey, United States, 2015

Vaccination, age group, increased-risk status, and race/ethnicity	Sample size	%	(95% CI)	Simple difference from 2014
**Influenza vaccination, 2014–15 season^§^**				
**≥19 yrs**				
**Total**	**31,897**	**44.8**	**(43.7–45.8)**	**1.6^¶^**
White	19,905	48.5	(47.2–49.8)	1.8
Black	4,159	37.7	(35.3–40.3)**	1.2
Hispanic or Latino	5,286	33.0	(30.9–35.2)**	-0.2
Asian	1,733	49.0	(44.8–53.3)	4.4
Other	814	43.0	(35.5–51.4)	4.4
**19–49 yrs**				
**Total**	**15,785**	**32.5**	**(31.1–33.9)**	**1.0**
White	8,635	34.6	(32.8–36.4)	1.8
Black	2,073	29.1	(26.1–32.4)**	-0.7
Hispanic or Latino	3,529	25.1	(22.7–27.7)**	-1.9
Asian	1,054	43.1	(37.6–49.1)	7.1
Other	494	32.2	(24.2–41.9)	-0.2
**50–64 yrs**				
**Total**	**8,296**	**48.7**	**(46.6–50.8)**	**1.0**
White	5,542	50.2	(47.7–52.7)	0.4
Black	1,166	41.9	(37.3–46.9)**	2.0
Hispanic or Latino	1,035	44.9	(38.2–52.3)	4.2
Asian	370	45.9	(37.5–55.2)	-5.7
Other	183	61.3	(46.1–76.7)	17.4
**≥65 yrs**				
**Total**	**7,816**	**73.5**	**(71.7–75.2)**	**2.0**
White	5,728	75.1	(73.2–77.0)	1.7
Black	920	64.3	(58.1–70.6)**	3.8
Hispanic or Latino	722	64.1	(57.9–70.4)**	0.1
Asian	309	83.5	(71.6–92.5)	11.0
Other	137	77.2	(65.0–87.6)	13.6
**Pneumococcal vaccination, ever^††^**				
**19–64 yrs, increased risk**				
**Total**	**8,196**	**23.0**	**(21.8–24.3)**	**2.8^¶^**
White	5,174	24.0	(22.5–25.6)	2.9^¶^
Black	1,225	22.0	(19.0–25.4)	1.8
Hispanic or Latino	1,206	19.4	(16.6–22.6)**	3.1
Asian	290	21.5	(15.4–29.2)	6.9
Other	301	22.6	(16.5–30.1)	-2.7
**≥65 yrs**				
**Total**	**8,057**	**63.6**	**(62.1–65.1)**	**2.3**
White	5,893	68.1	(66.4–69.9)	3.4^¶^
Black	946	50.2	(46.5–53.9)**	0.4
Hispanic or Latino	757	41.7	(37.2–46.3)**	-3.5
Asian	314	49.0	(41.7–56.3)**	1.3
Other	147	62.7	(51.0–73.0)	-6.7
**Tetanus vaccination (received in past 10 years)^§§^**				
**≥19 yrs**				
**Total**	**31,441**	**61.6**	**(60.7–62.5)**	**-0.6**
White	19,594	66.5	(65.3–67.6)	-0.8
Black	4,128	51.9	(49.8–54.0)**	1.3
Hispanic or Latino	5,186	51.9	(49.9–53.8)**	-0.3
Asian	1,705	51.0	(47.8–54.2)**	0.5
Other	828	63.8	(58.7–68.6)	-7.6^¶^
**19–49 yrs**				
**Total**	**15,369**	**62.1**	**(60.9–63.3)**	**-0.5**
White	8,420	68.5	(66.9–70.0)	-0.5
Black	2,021	53.4	(50.6–56.3)**	0.6
Hispanic or Latino	3,398	51.3	(49.1–53.6)**	-0.6
Asian	1,034	54.1	(50.2–58.0)**	2.3
Other	496	63.8	(57.3–69.7)	-8.2
**50–64 yrs**				
**Total**	**8,216**	**64.1**	**(62.6–65.5)**	**-0.6**
White	5,446	68.7	(67.0–70.4)	-0.7
Black	1,181	53.0	(49.4–56.6)**	3.2
Hispanic or Latino	1,041	54.5	(50.5–58.4)**	-0.1
Asian	363	45.2	(37.6–53.0)**	-3.9
Other	185	64.6	(53.3–74.5)	-10.3
**≥65 yrs**				
**Total**	**7,856**	**56.9**	**(55.3–58.4)**	**-0.9**
White	5,728	59.4	(57.6–61.1)	-1.3
Black	926	43.8	(39.5–48.1)**	0.6
Hispanic or Latino	747	50.6	(45.5–55.7)**	1.4
Asian	308	46.7	(39.9–53.7)**	0.1
Other	147	62.8	(47.4–76.0)	-0.2
**Tetanus vaccination including pertussis vaccine (received in past 10 years)^¶¶^**				
**≥19 yrs**				
**Total**	**20,406**	**23.1**	**(22.1–24.2)**	**3.1^¶^**
White	12,264	27.0	(25.7–28.4)	3.2^¶^
Black	2,820	15.1	(13.2–17.2)**	3.5^¶^
Hispanic or Latino	3,545	14.3	(12.7–16.0)**	1.8
Asian	1,233	19.9	(17.2–22.8)**	4.3^¶^
Other	544	29.7	(22.9–37.5)	2.3
Living with an infant aged <1 yr	608	41.9	(36.5–47.6)	10.0^¶^
Not living with an infant aged <1 yr	19,798	22.4	(21.4–23.4)***	2.8^¶^
**19–64 yrs**				
**Total**	**15,262**	**24.7**	**(23.6–25.9)**	**3.3^¶^**
White	8,627	29.7	(28.2–31.3)	3.6^¶^
Black	2,146	16.1	(14.0–18.4)**	3.4^¶^
Hispanic or Latino	3,031	14.8	(13.2–16.6)**	1.8
Asian	1,003	20.9	(17.9–24.2)**	5.3^¶^
Other	455	31.1	(24.1–39.1)	2.4
Living with an infant aged <1 yr	601	42.0	(36.6–47.6)	9.5^¶^
Not living with an infant aged <1 yr	14,661	23.9	(22.8–25.1)***	3.0^¶^
**≥65 yrs**				
**Total**	**5,144**	**16.5**	**(15.0–18.1)**	**2.5**
White	3,637	18.2	(16.4–20.1)	2.5
Black	674	9.7	(7.2–12.9)**	4.7^¶^
Hispanic or Latino	514	9.1	(6.0–13.6)**	2.5
Asian	230	13.8	(8.6–21.4)	-1.4
Other	89	—^†††^	—	—
Living with an infant aged <1 yr	7	—	—	—
Not living with an infant aged <1 yr	5,137	16.5	(14.9–18.1)	2.4
**Hepatitis A vaccination (at least 2 doses), ever^§§§^**				
**≥19 yrs**				
**Total**	**28,680**	**9.0**	**(8.5–9.5)**	**0.1**
Traveler^¶¶¶^	9,085	16.0	(15.0–17.0)	0.0
Nontraveler****	19,543	5.4	(5.0–5.9)^††††^	-0.1
With chronic liver conditions, overall	370	8.6	(5.6–13.0)	-5.2
**19–49 yrs**				
**Total**	**13,272**	**12.3**	**(11.5–13.2)**	**0.2**
White	7,314	12.8	(11.8–13.8)	0.0
Black	1,762	10.8	(8.9–13.0)	-0.4
Hispanic or Latino	2,948	10.4	(9.0–11.9)**	0.8
Asian	824	17.9	(14.5–21.8)**	2.7
Other	424	14.1	(9.9–19.8)	-0.6
Traveler	4,931	19.2	(17.8–20.8)	0.4
Nontraveler	8,321	8.1	(7.3–9.0) ^††††^	0.0
With chronic liver conditions, overall	101	—	—	—
**≥50 yrs**				
**Total**	**15,408**	**5.5**	**(5.0–6.0)**	**0.0**
Traveler	4,154	11.6	(10.4–13.0)	-0.3
Nontraveler	11,222	2.9	(2.4–3.4) ^††††^	0.0
With chronic liver conditions, overall	269	8.5	(4.9–14.4)	-3.8
**Hepatitis B vaccination (at least 3 doses), ever^§§§§^**				
**≥19 yrs**				
**Total**	**29,743**	**24.6**	**(23.8–25.4)**	**0.1**
Traveler	9,717	31.6	(30.4–32.9)	1.1
Nontraveler	19,964	20.9	(20.0–21.8)^††††^	-0.6
With chronic liver conditions, overall	378	27.4	(21.6–34.1)	-2.4
**19–49 yrs**				
**Total**	**14,076**	**32.0**	**(30.7–33.2)**	**-0.3**
White	7,726	34.9	(33.3–36.6)	-1.4
Black	1,881	29.4	(26.8–32.2)**	-0.5
Hispanic or Latino	3,085	22.5	(20.5–24.6)**	2.3
Asian	928	38.3	(34.6–42.1)	2.7
Other	456	36.2	(29.5–43.6)	2.7
Traveler	5,405	38.3	(36.5–40.1)	1.3
Nontraveler	8,647	28.0	(26.5–29.5)^††††^	-1.4
With chronic liver conditions, overall	107	30.0	(19.0–44.0)	-11.5
**≥50 yrs**				
**Total**	**15,667**	**16.5**	**(15.6–17.4)**	**0.8**
Traveler	4,312	22.3	(20.6–24.2)	1.2
Nontraveler	11,317	13.9	(13.0–14.9)^††††^	0.6
With chronic liver conditions, overall	271	26.3	(19.5–34.4)	1.1
**With diabetes, overall**				
19–59 yrs	1,195	24.4	(21.1–28.0)	0.8
≥60 yrs	2,075	12.6	(10.8–14.7)	-0.9
**Herpes zoster (shingles) vaccination, ever^¶¶¶¶^**				
**≥60 yrs**				
**Total**	**10,855**	**30.6**	**(29.3–31.9)**	**2.7^¶^**
White	7,832	34.6	(33.1–36.2)	2.7^¶^
Black	1,328	13.6	(11.1–16.6)**	2.0
Hispanic or Latino	1,055	16.0	(13.4–18.9)**	1.3
Asian	437	26.0	(20.4–32.5)**	9.5^¶^
Other	203	28.0	(18.8–39.5)	11.8
**60–64 yrs**				
**Total**	**2,729**	**21.7**	**(19.5–24.0)**	**1.3**
White	1,896	25.1	(22.3–28.0)	0.8
Black	379	12.7	(8.6–18.3)**	4.6
Hispanic or Latino	285	9.1	(5.8–13.9)**	-2.1
Asian	113	14.6	(8.5–23.9)**	6.8
Other	56	—	—	—
**≥65 yrs**				
**Total**	**8,126**	**34.2**	**(32.7–35.7)**	**3.2^¶^**
White	5,936	38.3	(36.5–40.0)	3.2^¶^
Black	949	14.1	(11.4–17.4)**	0.6
Hispanic or Latino	770	19.2	(16.1–22.9)**	2.9
Asian	324	30.6	(23.9–38.3)**	9.9^¶^
Other	147	28.9	(17.1–44.4)	9.3
HPV vaccination among females (at least 1 dose), ever*****				
**19–21 yrs**				
**Total**	**540**	**42.0**	**(36.3–47.9)**	**-2.8**
**22–26 yrs**				
**Total**	**1,261**	**41.4**	**(37.2–45.6)**	**3.8**
**19–26 yrs**				
**Total**	**1,801**	**41.6**	**(38.2–45.1)**	**1.3**
White	917	44.7	(39.9–49.5)	-1.6
Black	269	38.0	(29.7–47.1)	0.6
Hispanic or Latino	418	35.7	(29.9–42.0)**	7.7
Asian	108	36.3	(24.8–49.5)	13.5
Other	89	45.5	(29.9–62.1)	-1.8
HPV vaccination among males (at least 1 dose), ever*****				
**19–26 yrs**				
**Total**	**1,575**	**10.1**	**(8.3–12.3)**	**1.9**
**19–21 yrs**				
**Total**	**479**	**15.7**	**(12.0–20.5)**	**2.4**
**22–26 yrs**				
**Total**	**1,096**	**7.3**	**(5.4–9.6)**	**1.9**
**HPV vaccination (at least 1 dose) during ages 19–26 years, among females without reported HPV vaccination prior to age 19 years^†††††^**				
**Total**	**1,194**	**12.2**	**(9.8–15.2)**	**0.4**
White	560	11.8	(8.6–15.9)	-2.1
Black	188	8.6	(5.0–14.3)	-5.6
Hispanic or Latino	305	14.0	(8.6–21.9)	6.6
Asian	86	—	—	—
Other	55	—	—	—
**HPV vaccination (at least 1 dose) during ages 19–26 years, among males without reported HPV vaccination prior to age 19 years^†††††^**				
**Total**	**1,466**	**3.3**	**(2.2–4.9)**	**1.0**
White	791	3.8	(2.2–6.5)	1.9
Black	163	—	—	—
Hispanic or Latino	339	—	—	—
Asian	115	—	—	—
Other	58	—	—	—

**Abbreviations:** CI = confidence interval; HPV = human papillomavirus; Td = tetanus-diphtheria toxoid; Tdap = tetanus, diphtheria, and acellular pertussis vaccine.

* Adults were considered at increased risk for pneumococcal disease if they had ever been told by a doctor or other health professional that they had diabetes, emphysema, chronic obstructive pulmonary disease, coronary heart disease, angina, heart attack, or other heart condition; had a diagnosis of cancer during the previous 12 months (excluding nonmelanoma skin cancer); had ever been told by a doctor or other health professional that they had lymphoma, leukemia, or blood cancer; had been told by a doctor or other health professional that they had chronic bronchitis or weak or failing kidneys during the preceding 12 months; had an asthma episode or attack during the preceding 12 months; or were current smokers. For hepatitis A and hepatitis B vaccination, data were collected on selected respondent characteristics that increase the risk for infection (travel to countries where hepatitis A infections are endemic and having chronic liver disease; having diabetes, travel to countries where hepatitis B infections are endemic, and having chronic liver disease, respectively).

^†^ Race/ethnicity was categorized as follows: Hispanic, black, white, Asian and “other.” In this report, persons identified as Hispanic might be of any race. Persons identified as black, white, Asian, or other race are non-Hispanic. “Other” includes American Indian/Alaska Native and multiple race. The five racial/ethnic categories are mutually exclusive.

^§^ Respondents were asked if they had received an influenza shot or nasal spray in the past 12 months and if so, in which month and year. Missing month and year were imputed (3.5%) and interviews conducted during August 2014–June 2015 were used to estimate vaccination coverage during July 2014–May 2015 using Kaplan–Meier survival analysis. Differences were measured as the simple difference between the 2013–14 and 2014–15 influenza seasons.

^¶^ p<0.05 by T test for comparisons between 2015 and 2014 (overall and within each level of each characteristic).

** p<0.05 by T test for comparisons with non-Hispanic white as the reference group.

^††^ Respondents were asked if they had ever had a pneumonia shot.

^§§^ Respondents were asked if they had received a tetanus shot in the past 10 years. Vaccinated respondents included adults who received Td during the past 10 years or Tdap during 2005–2015.

^¶¶^ Respondents who had received a tetanus shot in the past 10 years were asked if their most recent shot was given in 2005 or later. Respondents who had received a tetanus shot since 2005 were asked if they were told that their most recent tetanus shot included the pertussis or whooping cough vaccine. Among 33,348 respondents aged ≥19 years, those without a "yes" or "no" classification for tetanus vaccination status within the preceding 10 years (n = 1,907 [5.7%]), for tetanus vaccination status during 2005–2015 (n = 591 [1.7%]), or those who reported tetanus vaccination during 2005–2015, but were not told vaccine type by the provider (n = 8,408 [25.2%]), did not know vaccine type (Td or Tdap) (n = 2,031 [6.1%]), or refused to answer or for whom data were not obtained (n = 5 [0.01%]) were excluded, yielding a sample of 20,406 respondents aged ≥19 years for whom Tdap vaccination status could be assessed. In February 2012, ACIP recommended Tdap vaccination for all adults aged ≥19 years, including adults aged ≥65 years.

*** p<0.05 by T test for comparisons between persons living with an infant aged <1 and persons not living with an infant aged <1 year.

^†††^ Estimate is not reliable due to small sample size (n<30) or relative standard error (standard error/estimates) >0.3.

^§§§^ Respondents were asked if they had ever received the hepatitis A vaccine, and if yes, were asked how many doses were received.

^¶¶¶^ Had traveled outside the United States to countries other than countries in Europe, Japan, Australia, New Zealand, or Canada since 1995.

**** Had not traveled outside the United States to countries other than countries in Europe, Japan, Australia, New Zealand, or Canada since 1995.

^††††^ p<0.05 by T test for comparisons between persons who had traveled outside the United States to countries other than countries in Europe, Japan, Australia, New Zealand, or Canada since 1995 and persons who had not traveled outside the United States to these areas since 1995.

^§§§§^ Respondents were asked if they had ever received the hepatitis B vaccine, and if yes, if they had received at least 3 doses or less than 3 doses.

^¶¶¶¶^ Respondents were asked if they had ever received a shingles vaccine.

***** Respondents were asked if they had ever received the HPV shot or cervical cancer vaccine, and if yes, age at the first dose.

^†††††^ The denominator includes persons aged 19–26 years without HPV vaccination prior to age 19 years, and the numerator includes those in the denominator who reported first HPV dose at age 19–26 years.

**TABLE 2:** Estimated proportion of health care personnel* who received selected vaccinations, by race/ethnicity^†^ — National Health Interview Survey, United States, 2015

Vaccination and race/ethnicity	Sample size	%	(95% CI)	Simple difference from 2014
**Influenza vaccination, 2014–15 season^§^**				
**≥19 yrs**				
**Total**	**2,636**	**68.6**	**(65.0–72.2)**	**3.2**
White	1,707	71.2	(66.8–75.4)	3.6
Black	404	59.8	(51.5–68.2)^¶^	9.5
Hispanic or Latino	294	60.0	(51.3–68.8)^¶^	-5.6
Asian	156	74.3	(58.0–88.0)	-3.4
Other	75	68.7	(49.7–85.9)	8.4
**19–49 yrs**				
**Total**	**1,593**	**65.5**	**(61.0–69.9)**	**3.8**
White	953	68.7	(62.7–74.5)	5.3
Black	277	56.9	(47.7–66.4)^¶^	8.9
Hispanic or Latino	208	57.4	(47.3–67.9)	-9.9
Asian	106	69.2	(49.0–87.2)	-6.1
Other	49	62.5	(40.0–84.8)	1.5
**50–64 yrs**				
**Total**	**743**	**71.7**	**(65.5–77.6)**	**0.6**
White	521	72.1	(65.3–78.5)	0.0
Black	105	63.3	(49.3–77.2)	4.8
Hispanic or Latino	69	69.3	(50.2–86.5)	-8.6
Asian	33	88.5	(67.5–98.4)	9.7
Other	15	—**	—	—
**≥65 yrs**				
**Total**	**300**	**82.2**	**(73.3–89.5)**	**7.0**
White	233	83.9	(73.5–91.9)	4.3
Black	22	—	—	—
Hispanic or Latino	17	—	—	—
Asian	17	—	—	—
Other	11	—	—	—
**Tetanus vaccination including pertussis vaccine, past 10 years^††^**				
**≥19 yrs**				
**Total**	**1,853**	**45.6**	**(42.6–48.7)**	**3.6**
White	1,208	49.2	(45.7–52.8)	2.8
Black	251	28.3	(21.5–36.3)^¶^	3.5
Hispanic or Latino	216	38.7	(30.3–47.7)^¶^	2.9
Asian	122	49.4	(38.3–60.6)	8.2
Other	56	56.8	(33.2–77.7)	17.3
**19–64 yrs**				
**Total**	**1,653**	**47.2**	**(44.0–50.6)**	**4.2**
White	1,056	51.5	(47.5–55.5)	3.7
Black	233	28.7	(21.7–37.0)^¶^	3.7
Hispanic or Latino	198	40.3	(31.6–49.7)^¶^	3.9
Asian	114	49.0	(37.6–60.5)	7.0
Other	52	57.5	(33.2–78.7)	17.9
**≥65 yrs**				
**Total**	**200**	**26.7**	**(19.0–36.2)**	**-2.0**
White	152	27.1	(18.6–37.8)	-2.5
Black	18	—	—	—
Hispanic or Latino	18	—	—	—
Asian	8	—	—	—
Other	4	—	—	—
**Hepatitis B vaccination (at least 3 doses), ever^§§^**				
**≥19 yrs**				
**Total**	**2,571**	**64.7**	**(62.2–67.2)**	**4.1^¶¶^**
White	1,663	67.8	(64.5–70.9)	4.8
Black	402	56.7	(51.4–62.0)^¶^	5.4
Hispanic or Latino	286	57.1	(48.6–65.1)^¶^	5.9
Asian	147	64.2	(54.0–73.2)	-4.1
Other	73	63.5	(44.9–78.8)	2.7

**Abbreviations:** CI = confidence interval; Td = tetanus-diphtheria toxoid; Tdap = tetanus, diphtheria, and acellular pertussis vaccine.

* Adults were classified as health care personnel if they reported they currently volunteer or work in a hospital, medical clinic, doctor’s office, dentist’s office, nursing home or some other health care facility including part-time and unpaid work in a health care facility as well as professional nursing care provided in the home.

^†^ Race/ethnicity was categorized as follows: Hispanic, black, white, Asian and “other.” In this report, persons identified as Hispanic might be of any race. Persons identified as black, white, Asian, or other race are non-Hispanic. “Other” includes American Indian/Alaska Native and multiple race. The five racial/ethnic categories are mutually exclusive.

^§^ Respondents were asked if they had received an influenza shot or nasal spray in the past 12 months and if so, in which month and year. Missing month and year were imputed (3.5%), and interviews conducted during August 2014–June 2015 were used to estimate vaccination coverage during July 2014–May 2015 using Kaplan-Meier survival analysis. Differences were measured as the simple difference between the 2013–14 and 2014–15 influenza seasons.

^¶^ p<0.05 by T test for comparisons with non-Hispanic white as the reference group.

** Estimate is not reliable due to small sample size (n<30) or relative standard error (standard error/estimates) >0.3.

^††^ Respondents who had received a tetanus shot in the past 10 years were asked if their most recent shot was given in 2005 or later. Respondents who had received a tetanus shot since 2005 were asked if they were told that their most recent tetanus shot included the pertussis or whooping cough vaccine. Among 2,729 health care personnel aged ≥19 years, those without a "yes" or "no" classification for tetanus vaccination status within the preceding 10 years (n = 66 [2.4%]), for tetanus vaccination status during 2005–2015 (n = 45 [1.6%]), or those who reported tetanus vaccination during 2005–2015, but were not told vaccine type by the provider (n = 595 [21.8%]) or did not know vaccine type (Td or Tdap) (n = 170 [6.2%]) were excluded, yielding a sample of 1,853 respondents aged ≥19 years for whom Tdap vaccination status could be assessed. In February 2012, ACIP recommended Tdap vaccination for all adults aged ≥19 years, including adults aged ≥65 years.

^§§^ Respondents were asked if they had ever received the hepatitis B vaccine, and if yes, if they had received at least 3 doses or less than 3 doses.

^¶¶^ p<0.05 by T test for comparisons between 2015 and 2014 within each level of each characteristic.

**TABLE 3:** Estimated proportion of health care personnel* with direct patient care responsibilities^†^ who received selected vaccinations, by race/ethnicity^§^ — National Health Interview Survey, United States, 2015

Vaccination, direct patient care responsibilities, and race/ethnicity	Sample size	%	(95% CI)	Simple difference from 2014
**Influenza vaccination, 2014–15 season^¶^**				
**≥19 yrs, with direct patient care responsibilities**				
**Total**	**1,630**	**68.9**	**(64.5–73.1)**	**3.8**
White	1,038	72.8	(67.5–77.9)	7.2
Black	275	53.8	(43.3–65.1)**	0.9
Hispanic or Latino	171	59.8	(49.7–70.2)**	-15.0
Asian	103	79.6	(60.0–93.6)	-0.2
Other	43	63.2	(40.2–85.7)	2.5
**≥19 yrs, without direct patient care responsibilities**				
**Total**	**1,006**	**67.9**	**(62.2–73.4)**	**1.9**
White	669	68.2	(61.7–74.5)	-2.5
Black	129	72.0	(61.5–81.7)^††^	28.1^§§^
Hispanic or Latino	123	60.2	(45.4–75.5)	7.6
Asian	53	65.8	(42.6–87.4)	-6.5
Other	32	80.5	(54.2–96.7)	^¶¶^
Tetanus vaccination including pertussis vaccine, past 10 years***				
**≥19 yrs, with direct patient care responsibilities**				
**Total**	**1,182**	**51.1**	**(47.2–54.9)**	**3.6**
White	768	55.0	(50.5–59.4)	2.6
Black	164	31.4	(22.6–41.8)**	2.6
Hispanic or Latino	134	44.1	(33.0–55.9)	0.6
Asian	78	55.2	(41.1–68.6)	10.1
Other	38	64.4	(35.2–85.8)	18.1
**≥19 yrs, without direct patient care responsibilities**				
**Total**	**671**	**35.5**	**(31.1–40.2)^††^**	**3.9**
White	440	38.8	(32.8–45.0)^††^	3.2
Black	87	22.2	(13.4–34.4)**	7.9
Hispanic or Latino	82	29.0	(20.2–39.6)^††^	6.1
Asian	44	38.5	(23.2–56.6)	4.9
Other	18	—^†††^	—	—
**Hepatitis B vaccination (at least 3 doses), ever^§§§^**				
**≥19 yrs, with direct patient care responsibilities**				
**Total**	**1,584**	**74.1**	**(71.4–76.7)**	**6.4^§§^**
White	1,009	78.2	(74.9–81.1)	7.2^§§^
Black	258	62.4	(55.7–68.7)**	5.8
Hispanic or Latino	172	70.0	(60.6–78.0)	10.7
Asian	96	72.3	(59.1–82.4)	3.1
Other	49	64.6	(42.7–81.7)	-5.7
**≥19 yrs, without direct patient care responsibilities**				
**Total**	**987**	**49.3**	**(44.8–53.8)^††^**	**1.7**
White	654	51.7	(45.8–57.6)^††^	2.7
Black	144	46.0	(35.7–56.6)^††^	7.6
Hispanic or Latino	114	37.9	(26.9–50.3)**^††^	-0.8
Asian	51	48.2	(31.2–65.6)^††^	-18.0
Other	24	—	—	—

**Abbreviations:** CI = confidence interval; HCP = health care personnel; Td = tetanus-diphtheria toxoid; Tdap = tetanus, diphtheria, and acellular pertussis vaccine.

* Adults were classified as HCP if they reported that they currently volunteer or work in a hospital, medical clinic, doctor’s office, dentist’s office, nursing home or some other health care facility including part-time and unpaid work in a health care facility as well as professional nursing care provided in the home.

^†^ HCP were classified as having direct patient care if they reported providing direct patient care (physical or hands on contact with patients) as part of their routine work.

^§^ Race/ethnicity was categorized as follows: Hispanic, black, white, Asian and “other.” In this report, persons identified as Hispanic might be of any race. Persons identified as black, white, Asian, or other race are non-Hispanic. “Other” includes American Indian/Alaska Native and multiple race. The five racial/ethnic categories are mutually exclusive.

^¶^ Respondents were asked if they had received an influenza shot or nasal spray in the past 12 months and if so, in which month and year. Missing month and year were imputed (3.5%), and interviews conducted during August 2014–June 2015 were used to estimate vaccination coverage during July 2014–May 2015 using Kaplan-Meier survival analysis. Differences were measured as the simple difference between the 2013–14 and 2014–15 influenza seasons.

** p<0.05 by T test for comparisons with non-Hispanic white as the reference group.

^††^ p<0.05 by T test for comparisons between HCP with direct patient care responsibilities and HCP without direct patient care responsibilities.

^§§^ p<0.05 by T test for comparisons between 2015 and 2014 within each level of each characteristic.

^¶¶^ Difference could not be estimated because estimate from previous season was suppressed.

*** Respondents who had received a tetanus shot in the past 10 years were asked if their most recent shot was given in 2005 or later. Respondents who had received a tetanus shot since 2005 were asked if they were told that their most recent tetanus shot included the pertussis or whooping cough vaccine. Among 2,729 health care personnel aged ≥19 years, those without a "yes" or "no" classification for tetanus vaccination status within the preceding 10 years (n = 66 [2.4%]), for tetanus vaccination status during 2005–2015 (n = 45 [1.6%]), or those who reported tetanus vaccination during 2005–2015, but were not told vaccine type by the provider (n = 595 [21.8%]) or did not know vaccine type (Td or Tdap) (n = 170 [6.2%]) were excluded, yielding a sample of 1,853 respondents aged ≥19 years for whom Tdap vaccination status could be assessed. In February 2012, ACIP recommended Tdap vaccination for all adults aged ≥19 years, including adults aged ≥65 years.

^†††^ Estimate is not reliable due to small sample size (n<30) or relative standard error (standard error/estimates) >0.3.

^§§§^ Respondents were asked if they had ever received the hepatitis B vaccine, and if yes, if they had received at least 3 doses or less than 3 doses.

### Pneumococcal Vaccination Coverage

Reported pneumococcal vaccination coverage (23-valent pneumococcal polysaccharide vaccine [PPSV23] and 13-valent pneumococcal conjugate vaccine [PCV13]) among adults aged 19–64 years at increased risk for pneumococcal disease was 23.0% overall, a 2.8 percentage point increase from 2014 (Table 1). Coverage among whites aged 19–64 years at increased risk was higher (24.0%) compared with Hispanics (19.4%) but did not differ for other racial/ethnic groups compared with whites. Among adults aged ≥65 years, coverage was 63.6% overall, similar to the estimate for 2014. Coverage among whites aged ≥65 years (68.1%) was higher compared with blacks (50.2%), Hispanics (41.7%), and Asians (49.0%) (Table 1). During 2010–2015, pneumococcal vaccination coverage among adults aged 19–64 years at increased risk and adults aged ≥65 years ranged from 18.5%–23.0% to 59.7%–63.6%, respectively, representing increases in coverage for both age groups (test for trend: p< 0.01 for persons aged 19–64 years and p = 0.01 for persons aged ≥65 years) (see https://stacks.cdc.gov/view/cdc/44995).

### Tetanus Vaccination Coverage

In 2015, the proportion of adults reporting having received any tetanus toxoid-containing vaccination during the past 10 years was 61.6% overall for adults aged ≥19 years, 62.1% for adults aged 19–49 years, 64.1% for adults aged 50–64 years, and 56.9% for adults aged ≥65 years (Table 1). The proportion of adults receiving tetanus vaccination during the past 10 years across all age groups did not change compared with 2014. Whites had higher coverage across all age groups compared with blacks, Hispanics, and Asians. During 2010–2015, tetanus vaccination among adults aged ≥19 years was unchanged at approximately 62% (see https://stacks.cdc.gov/view/cdc/44994).

Among adults aged ≥19 years for whom Tdap vaccination specifically could be assessed, overall reported coverage in the past 10 years was 23.1%, a 3.1 percentage point increase compared with 2014 (Table 1). Tdap coverage for black (15.1%), Hispanic (14.3%), and Asian (19.9%) adults aged ≥19 years was lower compared with whites (27.0%). Coverage among adults aged ≥19 years who reported living with an infant aged <1 year* was 41.9%, a 10 percentage point increase compared with the 2014 estimate. This was higher than the 22.4% coverage among adults aged ≥19 years without household contact with an infant aged <1 year, although Tdap coverage in this group increased 2.8 percentage points compared with 2014. During 2010–2015, Tdap vaccination coverage increased from 8.2% to 24.7% among adults aged 19–64 years (test for trend: p<0.01), and during 2012–2015 increased from 8.0% to 16.5% among adults aged ≥65 years (test for trend: p<0.01) (see https://stacks.cdc.gov/view/cdc/44994). Among 16,996 respondents who reported receiving a tetanus vaccination during 2005–2015, almost half (49.1%) reported that they were not informed of the vaccination type, and 12.6% could not recall what type of tetanus vaccination they had received (Table 4). Of the remaining 38.3% of respondents who reported that they knew what type of tetanus vaccine they received, 72.0% reported receiving Tdap.

**TABLE 4:** Type of tetanus vaccine received, and proportion that were tetanus, diphtheria, acellular pertussis vaccine, among adults aged ≥19 years, by selected characteristics — National Health Interview Survey, United States, 2015

Characteristic	Type of tetanus toxoid-containing vaccine received during 2005–2015									Proportion that was Tdap of the total tetanus toxoid-containing vaccine during 2005–2015*		
	No. in sample	Received Tdap		Received other tetanus vaccine		Doctor did not inform the patient		Could not recall vaccine type				
		%	(95% CI)	%	(95% CI)	%	(95% CI)	%	(95% CI)	No. in sample	%	(95% CI)
**Age group (yrs)**												
**≥19**												
**Total**	16,996	27.6	(26.5–28.8)	10.8	(10.0–11.5)	49.1	(47.7–50.5)	12.6	(11.7–13.5)	6,550	72.0	(70.2–73.7)
HCP^†^	1,853	45.1	(42.1–48.2)	12.6	(10.7–14.8)	32.0	(28.9–35.2)	10.3	(8.3–12.8)	1,087	78.2^§^	(74.6–81.3)
Non-HCP	15,124	25.5	(24.3–26.7)	10.5	(9.8–11.4)	51.2	(49.7–52.6)	12.8	(11.9–13.7)	5,460	70.8	(68.7–72.7)
**19–64**												
**Total**	13,224	28.9	(27.6–30.2)	10.6	(9.9–11.5)	47.8	(46.3–49.4)	12.7	(11.7–13.7)	5,295	73.1	(71.2–74.9)
HCP	1,658	46.3	(43.1–49.7)	12.5	(10.5–14.8)	31.0	(27.8–34.4)	10.2	(8.0–12.8)	998	78.8^§^	(75.0–82.1)
Non-HCP	11,548	26.4	(25.1–27.8)	10.4	(9.5–11.3)	50.2	(48.6–51.8)	13.0	(12.0–14.0)	4,294	71.8	(69.6–73.9)
**≥65**												
**Total**	3,772	21.9	(20.0–23.9)	11.3	(9.9–12.8)	54.8	(52.5–57.2)	12.0	(10.5–13.6)	1,255	65.9	(62.2–69.4)
HCP	195	29.1	(20.8–39.2)	14.2	(8.3–23.1)	44.5	(35.4–54.0)	12.1	(7.0–20.1)	89	67.2	(50.8–80.3)
Non-HCP	3,576	21.5	(19.6–23.6)	11.2	(9.8–12.8)	55.3	(52.9–57.7)	12.0	(10.5–13.6)	1,166	65.8	(62.0–69.5)

**Abbreviations:** CI = confidence interval; HCP = health care personnel; Td = tetanus-diphtheria toxoid; Tdap = tetanus, diphtheria, and acellular pertussis vaccine

* Calculated by dividing the number of respondents who reported receiving Tdap by the sum of those who reported receiving Tdap and those who reported receiving other tetanus vaccination; respondents who reported that the doctor did not inform them of the vaccine type they received and those who could not recall the vaccine type were excluded.

^†^ Adults were classified as HCP if they reported they currently volunteer or work in a hospital, medical clinic, doctor’s office, dentist’s office, nursing home or some other health care facility including part-time and unpaid work in a health care facility as well as professional nursing care provided in the home.

^§^ p<0.05 by T test for comparisons between HCP and non-HCP.

Overall Tdap vaccination of HCP aged ≥19 years reported in 2015 was 45.6%, similar to the estimate from 2014 (Table 2). White HCP had higher Tdap coverage (49.2%) compared with black HCP (28.3%) and Hispanic HCP (38.7%). Among HCP aged ≥19 years with direct patient care responsibilities, Tdap vaccination coverage was 51.1%, similar to the 2014 estimate (Table 3). Black HCP with direct patient care responsibilities had lower Tdap coverage (31.4%) compared with white HCP (55.0%), but coverage for HCP in the other racial/ethnic groups was similar to that for white HCP (Table 3). Tdap vaccination among HCP aged 19–64 years increased from 22.0% in 2010 to 47.2% in 2015 (test for trend: p<0.01). Tdap vaccination among HCP aged ≥65 years reported during 2012–2015 ranged from 16.9% to 30.7% (see https://stacks.cdc.gov/view/cdc/44994). Among adults aged ≥19 years who received a tetanus vaccination and reported that they knew what type of tetanus vaccine they received, HCP reported receipt of Tdap (78.2%) more often than did non-HCP (70.8%) (Table 4).

### Hepatitis A Vaccination Coverage

In 2015, reported hepatitis A vaccination coverage (≥2 doses) was 9.0% for adults aged ≥19 years, 12.3% among adults aged 19–49 years, and 5.5% among adults aged ≥50 years, similar to the estimates for 2014 (Table 1). Among adults aged 19–49 years, coverage for Hispanics (10.4%) was lower than that for whites (12.8%); coverage for Asians (17.9%) was higher than that for whites. Vaccination coverage was higher among adults aged ≥19 years who had traveled outside the United States since 1995 to a country in which hepatitis A is of high or intermediate endemicity (countries other than the countries of Europe, Japan, Australia, New Zealand, or Canada) than among respondents who did not travel outside the United States or had traveled only to countries in which the disease is of low endemicity (16.0% versus 5.4%, respectively). Vaccination coverage among adult travelers to countries with high or intermediate endemicity was similar to the estimate for 2014. Overall coverage among adults aged ≥19 years with chronic liver conditions was 8.6%, similar to the 2014 estimate (Table 1). During 2010–2015 among all adults aged ≥19 years, hepatitis A vaccination coverage increased (range: 8.1%–9.1%; test for trend: p = 0.04), but remained stable among travelers to countries with high or intermediate endemicity, among nontravelers, and among persons with chronic liver conditions (see https://stacks.cdc.gov/view/cdc/44993).

### Hepatitis B Vaccination Coverage

Reported hepatitis B vaccination coverage (≥3 doses) among adults was 24.6% for adults aged ≥19 years, 32.0% among adults aged 19–49 years, and 16.5% among adults aged ≥50 years. Overall vaccination coverage among adults aged ≥19 years was similar to the 2014 estimate (Table 1). Vaccination coverage was higher among adults aged ≥19 years who had traveled outside the United States since 1995 to a country in which hepatitis B is of high or intermediate endemicity (countries other than the countries of Europe, Japan, Australia, New Zealand, or Canada) than among respondents who did not travel outside the United States or had traveled only to countries in which hepatitis B is of low endemicity (31.6% versus 20.9%, respectively). Among adults aged 19–49 years, vaccination coverage was lower for blacks (29.4%) and Hispanics (22.5%) compared with whites (34.9%). Overall coverage among adults aged ≥19 years with chronic liver conditions was 27.4%, similar to the 2014 estimate. Vaccination coverage for persons with diabetes was 24.4% for those aged 19–59 years and 12.6% for those aged ≥60 years, similar to estimates for 2014. Overall, hepatitis B vaccination coverage among HCP aged ≥19 years was 64.7%, a 4.1 percentage point increase compared with the estimate for 2014. Black (56.7%) and Hispanic HCP (57.1%) had lower coverage compared with white HCP (67.8%) (Table 2). Among HCP aged ≥19 years with direct patient care responsibilities, hepatitis B vaccination coverage was 74.1%, a 6.4 percentage point increase compared with the 2014 estimate (Table 3). Coverage for black HCP aged ≥19 years with direct patient care responsibilities was lower (62.4%) than that for white HCP with direct patient care responsibilities (78.2%) (Table 3). During 2010–2015, hepatitis B vaccination coverage decreased overall among adults aged ≥19 years, travelers to areas of high or intermediate endemicity aged ≥19 years, and nontravelers aged ≥19 years (range: 24.5%–27.1%, 30.5%–35.0%, and 20.9%–23.2%, respectively; test for trend: p<0.01 for all groups). Hepatitis B vaccination remained stable among adults aged ≥19 years with chronic liver conditions and among HCP aged ≥19 years (see https://stacks.cdc.gov/view/cdc/44993).

### Herpes Zoster Vaccination Coverage

In 2015, among adults aged ≥60 years, 30.6% reported receiving herpes zoster vaccination to prevent shingles, a 2.7 percentage point increase from 2014 (Table 1). Whites aged ≥60 years had higher herpes zoster vaccination coverage (34.6%) compared with blacks (13.6%), Hispanics (16.0%), and Asians (26.0%). Among adults aged 60–64 years, 21.7% reported receiving herpes zoster vaccination, with blacks (12.7%), Hispanics (9.1%), and Asians (14.6%) reporting lower coverage compared with that for whites (25.1%), similar to 2014 estimates. Among adults aged ≥65 years, 34.2% reported herpes zoster vaccination, a 3.2 percentage point increase from 2014. Whites aged ≥65 years had higher herpes zoster vaccination coverage (38.3%) compared with blacks (14.1%), Hispanics (19.2%), and Asians (30.6%). Herpes zoster vaccination among adults aged ≥60 years increased from 14.4% in 2010 to 30.6% in 2015 (test for trend: p<0.01) (see https://stacks.cdc.gov/view/cdc/44992).

### HPV Vaccination Coverage

In 2015, among women aged 19–26 years, 41.6% reported receipt of at least 1 dose of HPV vaccine, similar to the estimate reported for 2014 (Table 1). Coverage among women was similar by age group (42.0% and 41.4%, respectively, for women aged 19–21 and 22–26 years), and did not change for either age group from 2014 estimates. Among women aged 19–26 years, Hispanics (35.7%) had lower coverage compared with whites (44.7%), but coverage for blacks (38.0%), Asians (36.3%), and adults who indicated other race (45.5%) was similar to that for whites. Receipt of at least 1 dose of HPV vaccine among males aged 19–26 years was 10.1%, similar to the 2014 estimate. Coverage was 15.7% for males aged 19–21 years and 7.3% for those aged 22–26 years, similar to the 2014 estimates.

Among women aged 19–26 years, 2.1% reported receiving the first dose of HPV vaccine at age 8–10 years, 6.2% at age 11–12 years, 56.1% at age 13–17 years, 16.1% at age 18 years, and 19.6% at age 19–26 years (Table 5). Among males aged 19–26 years, 4.1% reported receiving the first dose of HPV vaccine at age 8–10 years, 6.9% at age 11–12 years, 43.3% at age 13–17 years, 15.4% at age 18 years, and 30.2% at age 19–26 years. Among respondents aged 19–26 years, the difference between the age reported at the time of the interview and the age at which respondents indicated that the first dose of HPV vaccine was received was ≥11 years for 6.1% of women and for 6.2% of males. This would imply receipt of vaccination in 2004 or earlier, before HPV vaccine was licensed for use in 2006. Among females and males aged 19–26 years who had not received HPV vaccine prior to age 19 years, 12.2% and 3.3% reported receiving the first dose of HPV vaccine at age 19–26 years, respectively. (Table 1). HPV vaccination increased from 20.7% in 2010 to 41.6% in 2015 for females aged 19–26 years, and from 2.1% in 2011 to 10.1% in 2015 among males aged 19–26 years (test for trend: p<0.01 for both groups) (see https://stacks.cdc.gov/view/cdc/44992).

**TABLE 5:** Age at first dose of human papillomavirus vaccination* and difference between age at interview^†^ among adults aged 19–26 years — National Health Interview Survey, United States, 2015

Characteristic	Females (N = 760)		Males (N = 162)	
	No.	Weighted %	No.	Weighted %
**Age at first dose (yrs)**				
**8–10**	**16**	**2.1**	**10**	**4.1**
8	4	0.5	6	2.9
9	2	0.1	0	0.0
10	10	1.4	4	1.2
**11–12**	**49**	**6.2**	**9**	**6.9**
11	7	1.2	4	3.0
12	42	5.0	5	4.0
**13–17**	**433**	**56.1**	**62**	**43.3**
13	66	7.6	6	2.9
14	68	10.6	6	3.6
15	90	11.1	13	6.2
16	130	17.3	20	15.9
17	79	9.4	17	14.7
**18**	**108**	**16.1**	**28**	**15.4**
**19–26**	**154**	**19.6**	**53**	**30.2**
19	43	5.3	11	7.8
20	39	4.8	13	7.9
21	27	3.5	11	4.4
22	14	1.4	9	2.2
23	14	2.5	3	2.2
24	11	1.6	4	4.5
25	3	0.3	2	1.2
26	3	0.2	0	0.0
**Difference between age at interview and age at first dose (yrs)**				
0	14	1.8	5	3.2
1	36	7.1	19	13.9
2	41	6.1	18	10.4
3	48	6.2	21	16.5
4	84	11.0	29	18.1
5	69	10.5	10	5.3
6	90	10.8	12	9.7
7	104	12.8	13	5.0
8	105	12.8	6	3.0
9	76	8.8	7	3.0
10	48	6.1	10	5.7
11	22	2.7	3	1.6
12	10	1.5	4	2.0
13	6	1.2	4	1.9
14	5	0.6	1	0.7
15	1	0.1	0	0.0
16	0	0.0	0	0.0
17	1	0.0	0	0.0

**Abbreviation:** HPV = human papillomavirus.

* Respondents were asked, "How old were you when you received your first HPV shot?"

^†^ The simple difference between age reported at time of interview and age the respondent indicated the first dose of the HPV vaccine was received. A difference of "zero" indicates that a respondent's reported age at first dose was the same as their age at interview.

### Racial and Ethnic Vaccination Differences

Compared with 2014, racial/ethnic differences in vaccination coverage persisted for all seven vaccines in this report and widened for pneumococcal and herpes zoster vaccination (due primarily to increases among whites) (Table 1**)**. Blacks, Hispanics, and Asians had lower vaccination coverage than that of whites for all of the vaccines routinely recommended for adults, except for: influenza vaccination (adults aged ≥19 years: Asians had coverage similar to whites; aged 50–64 years: Hispanics and Asians had coverage similar to whites; aged ≥65 years: Asians had coverage similar to whites); pneumococcal vaccination (adults aged 19–64 years with increased risk: blacks and Asians had coverage similar to whites); Tdap vaccination (adults aged ≥65 years: Asians had coverage similar to whites); hepatitis A vaccination (adults aged 19–49 years: blacks had coverage similar to and Asians had coverage higher than whites); hepatitis B (adults aged 19–49 years: Asians had coverage similar to whites); and HPV vaccination (females aged 19–26 years: blacks and Asians had coverage similar to whites). 

With whites as the reference group, there were differences in vaccination coverage for 51 of the 66 comparisons by vaccine and age/target groups (not including comparisons of the “other” race/ethnic group) (Table 6). These vaccination differences ranged from -2.4 percentage points for Hispanics versus whites for hepatitis A vaccination among adults aged 19–49 years to -26.4 percentage points for Hispanics versus whites for pneumococcal vaccination among adults aged ≥65 years. 

**TABLE 6:** Summary table of racial/ethnic differences in vaccination coverage among adults aged ≥19 years, by age group and increased-risk status* — National Health Interview Survey, United States, 2015

Vaccination, age group, increased-risk status	% vaccinated, whites	Vaccination differences^†^, blacks	Vaccination differences, Hispanics	Vaccination differences, Asians	Vaccination differences, other
**Influenza vaccination, 2014–15 season^§^**					
≥19 yrs	48.5	-10.8^¶^	-15.5^¶^	0.5	-5.5
19–49 yrs	34.6	-5.5^¶^	-9.5^¶^	8.5	-2.4
50–64 yrs	50.2	-8.3^¶^	-5.3	-4.3	11.1
≥65 yrs	75.1	-10.8^¶^	-11.0^¶^	8.4	2.1
HCP,** ≥19 yrs	71.2	-11.4^¶^	-11.2^¶^	3.1	-2.5
**Pneumococcal vaccination, ever^††^**					
19–64 yrs, increased risk	24.0	-2.0	-4.6^¶^	-2.5	-1.5
≥65 yrs	68.1	-17.9^¶^	-26.4^¶^	-19.1^¶^	-5.5
**Tetanus vaccination (received in past 10 years)^§§^**					
≥19 yrs	66.5	-14.5^¶^	-14.6^¶^	-15.5^¶^	-2.7
19–49 yrs	68.5	-15.1^¶^	-17.2^¶^	-14.4^¶^	-4.7
50–64 yrs	68.7	-15.7^¶^	-14.3^¶^	-23.5^¶^	-4.1
≥65 yrs	59.4	-15.6^¶^	-8.8^¶^	-12.6^¶^	3.5
**Tetanus vaccination including pertussis vaccine (received in past 10 years)^¶¶^**					
≥19 yrs	27.0	-11.9^¶^	-12.8^¶^	-7.2^¶^	2.7
19–64 yrs	29.7	-13.7^¶^	-14.9^¶^	-8.9^¶^	1.3
≥65 yrs	18.2	-8.5^¶^	-9.1^¶^	-4.4	—***
HCP, ≥19 yrs	49.2	-20.9^¶^	-10.5^¶^	0.2	7.6
**Hepatitis A vaccination (at least 2 doses)^†††^**					
19–49 yrs	12.8	-2.0	-2.4^¶^	5.1^¶^	1.3
**Hepatitis B vaccination (at least 3 doses)^§§§^**					
19–49 yrs	34.9	-5.5^¶^	-12.4^¶^	3.4	1.3
HCP, ≥19 yrs	67.8	-11.0^¶^	-10.7^¶^	-3.6	-4.3
**Herpes zoster (shingles) vaccination, ever^¶¶¶^**					
≥60 yrs	34.6	-21.0^¶^	-18.6^¶^	-8.6^¶^	-6.7
60–64 yrs	25.1	-12.4^¶^	-16.0^¶^	-10.4^¶^	—
≥65 yrs	38.3	-24.1^¶^	-19.0^¶^	-7.6^¶^	-9.4
HPV vaccination among females (at least 1 dose), ever****					
19–26 yrs	44.7	-6.7	-9.0^¶^	-8.4	0.9

**Abbreviations:** HCP = health care personnel; HPV = human papillomavirus

* Adults were considered at increased risk for pneumococcal disease if they had ever been told by a doctor or other health professional that they had diabetes, emphysema, chronic obstructive pulmonary disease, coronary heart disease, angina, heart attack, or other heart condition; had a diagnosis of cancer during the previous 12 months (excluding nonmelanoma skin cancer); had ever been told by a doctor or other health professional that they had lymphoma, leukemia, or blood cancer; had been told by a doctor or other health professional that they had chronic bronchitis or weak or failing kidneys during the preceding 12 months; had an asthma episode or attack during the preceding 12 months; or were current smokers.

^†^ Percentage point difference in vaccination coverage compared with non-Hispanic whites as the reference group.

^§^ Respondents were asked if they had received an influenza shot or nasal spray in the past 12 months and if so, in which month and year. Missing month and year were imputed (3.5%) and interviews conducted during August 2014–June 2015 were used to estimate vaccination coverage during July 2014–May 2015 using Kaplan–Meier survival analysis.

^¶^ p<0.05 by T test for comparisons with non-Hispanic white as the reference group.

** Adults were classified as HCP if they reported that they currently volunteer or work in a hospital, medical clinic, doctor’s office, dentist’s office, nursing home or some other health care facility including part-time and unpaid work in a health care facility as well as professional nursing care provided in the home.

^††^ Respondents were asked if they had ever had a pneumonia shot.

^§§^ Respondents were asked if they had received a tetanus shot in the past 10 years. Vaccinated respondents included adults who received Td during the past 10 years or Tdap during 2005–2015.

^¶¶^ Respondents who had received a tetanus shot in the past 10 years were asked if their most recent shot was given in 2005 or later. Respondents who had received a tetanus shot since 2005 were asked if they were told that their most recent tetanus shot included the pertussis or whooping cough vaccine. Among 33,348 respondents aged ≥19 years, those without a "yes" or "no" classification for tetanus vaccination status within the preceding 10 years (n = 1,907 [5.7%]), for tetanus vaccination status during 2005–2015 (n = 591 [1.7%]), or those who reported tetanus vaccination during 2005–2015, but were not told vaccine type by the provider (n = 8,408 [25.2%]), did not know vaccine type (Td or Tdap) (n = 2,031 [6.1%]), or refused to answer or for whom data were not obtained (n = 5 [0.01%]) were excluded, yielding a sample of 20,406 respondents aged ≥19 years for whom Tdap vaccination status could be assessed. In February 2012, ACIP recommended Tdap vaccination for all adults aged ≥19 years, including adults aged ≥65 years.

*** Estimate is not reliable due to small sample size (n<30) or relative standard error (standard error/estimates) >0.3.

^†††^ Respondents were asked if they had ever received the hepatitis A vaccine, and if yes, were asked how many doses were received.

^§§§^ Respondents were asked if they had ever received the hepatitis B vaccine, and if yes, if they had received at least 3 doses or less than 3 doses.

^¶¶¶^ Respondents were asked if they had ever received a shingles vaccine.

**** Respondents were asked if they had ever received the HPV shot or cervical cancer vaccine, and if yes, age at the first dose.

Among HCP with direct patient care, influenza coverage among white HCP was higher (72.8%) than that for black (53.8) and Hispanic (59.8%) HCP (Table 3). Among all HCP, white HCP had higher Tdap coverage (49.2%) compared with black HCP (28.3%) and Hispanic HCP (38.7%) (Table 2). Black HCP with direct patient care responsibilities had lower Tdap coverage (31.4%) compared with white HCP (55.0%), but Tdap coverage for HCP with direct patient care responsibilities in the other racial/ethnic groups was similar to that for white HCP (Table 3). Black (56.7%) and Hispanic HCP (57.1%) had lower hepatitis B coverage compared with white HCP (67.8%) (Table 2). Among HCP aged ≥19 years with direct patient care responsibilities, hepatitis B coverage for black HCP was lower (62.4%) than that for white HCP (78.2%) (Table 3).

During 2010–2015, vaccination differences between whites and blacks increased for Tdap (adults aged 19–64 years), hepatitis A (adults aged 19–49 years), and herpes zoster vaccination (adults aged ≥60 years and ≥65 years) (test for trend: p<0.05) (Table 7). Among Hispanics, vaccination differences increased over this time period compared with whites for Td (adults aged 19–49 years), Tdap (adults aged ≥19 years and 19–64 years), hepatitis A (adults aged 19–49 years), hepatitis B (HCP aged ≥19 years), and herpes zoster vaccination (adults aged ≥60 years and ≥65 years) (test for trend: p<0.05). For Asians, vaccination differences increased over this time period compared with whites for Tdap (adults aged 19–64 years) (test for trend: p<0.05). Among persons reporting other race, compared with whites during this time period vaccination gaps widened for hepatitis A vaccination (adults aged 19–49 years) (test for trend: p<0.05). Vaccination differences between whites, blacks, Hispanics, and persons reporting other race for the other vaccines and age groups did not change during this period (Table 7).

**TABLE 7:** Average change in racial/ethnic percentage point differences* in vaccination coverage among adults aged >19 years compared with whites, by age group and increased-risk status^†^ — National Health Interview Survey, United States, 2010–2015

Vaccination, age group, increased-risk status	Black		Hispanic		Asian		Other	
	%	(95% CI)	%	(95% CI)	%	(95% CI)	%	(95% CI)
**Influenza vaccination, 2009–10 through 2014–15 season^§^**								
≥19 yrs	-0.1	(-1.1 to 1.0)	-0.4	(-1.2 to 0.5)	0.1	(-1.1 to 1.2)	-0.5	(-1.4 to 0.4)
19–49 yrs	0.3	(-1.3 to 1.9)	-0.7	(-1.9 to 0.6)	0.6	(-1.2 to 2.4)	-0.3	(-1.5 to 0.9)
50–64 yrs	-0.4	(-2.7 to 1.8)	0.6	(-0.7 to 1.9)	-1.6	(-4.1 to 0.9)	-0.2	(-4.7 to 4.3)
≥65 yrs	0	(-1.4 to 1.3)	0.1	(-1.3 to 1.4)	1.3	(-0.6 to 3.3)	2.6	(-0.6 to 5.7)
HCP,^ ¶^ ≥19 yrs	0.1	(-1.9 to 2.1)	0.2	(-2.3 to 2.7)	-1.1	(-4.3 to 2.1)	-3.9	(-10.5 to 2.6)
Pneumococcal vaccination, ever**								
19–64 yrs, increased risk	-0.5	(-1.6 to 0.6)	-0.2	(-1.6 to 1.1)	0.3	(-1.7 to 2.3)	-1.0	(-3.4 to 1.4)
≥65 yrs	0.3	(-0.9 to 1.4)	-0.1	(-2.1 to 1.9)	0.6	(-2.6 to 3.7)	0.8	(-5.8 to 7.5)
**Tetanus vaccination (received in past 10 yrs)^††^**								
≥19 yrs	-0.6	(-1.3 to 0.1)	-0.5	(-1.0 to -0.1)	0.5	(-0.5 to 1.4)	0.1	(-1.9 to 2.2)
19–49 yrs	-0.5	(-1.2 to 0.1)	-0.7	(-1.3 to -0.1)**^§§^**	0.6	(-0.2 to 1.4)	0.4	(-2.6 to 3.4)
50–64 yrs	-0.6	(-2.2 to 0.9)	0.4	(-0.5 to 1.2)	0	(-2.8 to 2.7)	0.1	(-2.2 to 2.3)
≥65 yrs	-0.5	(-2.3 to 1.3)	0.5	(-0.7 to 1.6)	1.3	(-0.6 to 3.1)	0.1	(-5.6 to 5.8)
**Tetanus vaccination including pertussis vaccine (received in past 10 yrs)^¶¶^**								
≥19 yrs	-2.3	(-5.7 to 1.0)	-1.8	(-2.3 to -1.3)**^§§^**	-2.3	(-5.6 to 1.1)	-0.8	(-3.1 to 1.5)
19–64 yrs	-2.6	(-3.5 to -1.7)**^§§^**	-2.2	(-2.4 to -2.0)**^§§^**	-2.2	(-3.3 to -1.0)**^§§^**	0.4	(-1.6 to 2.4)
≥65 yrs	-2.3	(-7.3 to 2.7)	-1.4	(-3.4 to 0.5)	0.8	(-3.0 to 4.5)	0.2	(-3.5 to 4.0)
HCP, ≥19 yrs	-4.4	(-13.7 to 4.9)	-0.9	(-2.7 to 1.0)	-1.7	(-16.0 to 12.6)	-4.3	(-23.7 to 15.1)
Hepatitis A vaccination (at least 2 doses)***								
19–49 yrs	-0.4	(-0.6 to -0.2)**^§§^**	-0.5	(-0.9 to -0.2)**^§§^**	-0.5	(-1.7 to 0.7)	-1.3	(-2.4 to -0.3)**^§§^**
**Hepatitis B vaccination (at least 3 doses)^†††^**								
19–49 yrs	-0.8	(-1.7 to 0.1)	-1.0	(-2.3 to 0.3)	0	(-1.3 to 1.3)	-1.1	(-3.2 to 1.0)
HCP, ≥19 yrs	-1.2	(-3.2 to 0.7)	-1.2	(-2.3to -0.0)**^§§^**	-1.7	(-3.7 to 0.3)	-2.9	(-7.3 to 1.4)
**Herpes zoster (shingles) vaccination, ever^§§§^**								
≥60 yrs	-2.1	(-3.4 to -0.8)**^§§^**	-1.6	(-3.0 to -0.3)**^§§^**	-2.0	(-4.4 to 0.4)	-0.6	(-3.9 to 2.6)
60–64 yrs	-1.7	(-4.8 to 1.4)	-1.7	(-3.8 to 0.3)	-2.3	(-4.9 to 0.3)	0.5	(-4.0 to 5.0)
≥65 yrs	-2.3	(-2.9 to -1.6)**^§§^**	-1.5	(-2.9 to -0.2)**^§§^**	-1.6	(-4.2 to 0.9)	-1.4	(-4.0 to 1.1)
**HPV vaccination among females (at least 1 dose), ever^¶¶¶^**								
19–26 yrs	-1.4	(-4.4 to 1.5)	-0.8	(-5.9 to 4.2)	-2.8	(-10.6 to 5.0)	1.0	(-2.1 to 4.0)

**Abbreviation:** HCP = health care personnel.

* Estimated slope from weighted linear regression of percentage point difference in vaccination coverage between a racial/ethnic group and non-Hispanic whites on survey year/influenza season. For influenza, interviews from August through June of each season were used to estimate coverage from July through May using Kaplan Meier survival analysis. Tdap vaccination coverage data among adults aged ≥65 years are available beginning in the NHIS 2012 survey.

^†^ Adults were considered at increased risk for pneumococcal disease if they had ever been told by a doctor or other health professional that they had diabetes, emphysema, chronic obstructive pulmonary disease (beginning in 2012), coronary heart disease, angina, heart attack, or other heart condition; had a diagnosis of cancer during the previous 12 months (excluding nonmelanoma skin cancer); had ever been told by a doctor or other health professional that they had lymphoma, leukemia, or blood cancer; had been told by a doctor or other health professional that they had chronic bronchitis or weak or failing kidneys during the preceding 12 months; had an asthma episode or attack during the preceding 12 months; or they were current smokers.

^§^ Respondents were asked if they had received an influenza shot or nasal spray in the past 12 months and if so, in which month and year. Interviews from August through June of each season were used to estimate coverage from July through May using Kaplan Meier survival analysis.

^¶^ Adults were classified as HCP if they reported that they currently volunteer or work in a hospital, medical clinic, doctor’s office, dentist’s office, nursing home or some other health care facility including part-time and unpaid work in a health care facility as well as professional nursing care provided in the home.

** Respondents were asked if they had ever had a pneumonia shot.

^§§^ p<0.05 by linear trend test.

^††^ Respondents were asked if they had received a tetanus shot in the past 10 years. Vaccinated respondents included adults who received Td during the past 10 years or Tdap during 2005–2015.

^¶¶^ Respondents who had received a tetanus shot in the past 10 years were asked if their most recent shot was given in 2005 or later. Respondents who had received a tetanus shot since 2005 were asked if they were told that their most recent tetanus shot included the pertussis or whooping cough vaccine. Among 33,348 respondents aged ≥19 years, those without a "yes" or "no" classification for tetanus vaccination status within the preceding 10 years (n = 1,907 [5.7%]), for tetanus vaccination status during 2005–2015 (n = 591 [1.7%]), or those who reported tetanus vaccination during 2005–2015, but were not told vaccine type by the provider (n = 8,408 [25.2%]), did not know vaccine type (Td or Tdap) (n = 2,031 [6.1%]), or refused to answer or for whom data were not obtained (n = 5 [0.01%]) were excluded, yielding a sample of 20,406 respondents aged ≥19 years for whom Tdap vaccination status could be assessed. In February 2012, ACIP recommended Tdap vaccination for all adults aged ≥19 years, including adults aged ≥65 years.

*** Respondents were asked if they had ever received the hepatitis A vaccine, and if yes, were asked how many doses were received.

^†††^ Respondents were asked if they had ever received the hepatitis B vaccine, and if yes, if they had received at least 3 doses or less than 3 doses.

^§§§^ Respondents were asked if they had ever received a shingles vaccine.

^¶¶¶^ Respondents were asked if they had ever received the HPV shot or cervical cancer vaccine, and if yes, age at the first dose.

## Discussion

In 2015, adult vaccination coverage in the United States remained similar to 2014, except for modest increases in influenza (adults aged ≥19 years; for influenza coverage, increase compared with the 2013–14 season), pneumococcal (adults aged 19–64 years with increased risk), Tdap (adults aged ≥19 years), herpes zoster (adults aged ≥60 years) and hepatitis B (HCP aged ≥19 years). Overall, although the point estimates for each year varied by only a few percentage points, linear trend tests indicated that during 2010–2015, vaccination coverage increased for influenza and pneumococcal vaccines (all age and risk groups), Tdap (adults aged 19–64 years), hepatitis A (adults aged ≥19 years), herpes zoster (adults aged ≥60 years), and HPV (females aged 19–26 years) vaccines and during 2012–2015 and 2011–2015, for Tdap vaccine (adults aged ≥65 years) and HPV vaccine (males aged 19–26 years), respectively. Although these increases were small, collectively they might have resulted in meaningful reductions in disease among adults (*11*,*12*). During 2010–2015 there was a decreasing trend observed for hepatitis B vaccination among persons aged ≥19 years overall (Figure). Vaccination coverage estimates for three of the four vaccines in this report that are included in *Healthy People 2020* (influenza, pneumococcal, and hepatitis B [for HCP] vaccines) were below the respective target levels, including among insured adults and adults with multiple health care visits in the past year. Herpes zoster vaccination coverage in 2015 was 0.6 percentage points above the *Healthy People 2020* target of 30%. Racial/ethnic differences in vaccination coverage persisted for all seven vaccines discussed in this report. These data indicate multiple missed opportunities for vaccination and the need to increase routine assessment of adult vaccination needs, and vaccination with needed vaccines.

**FIGURE:**
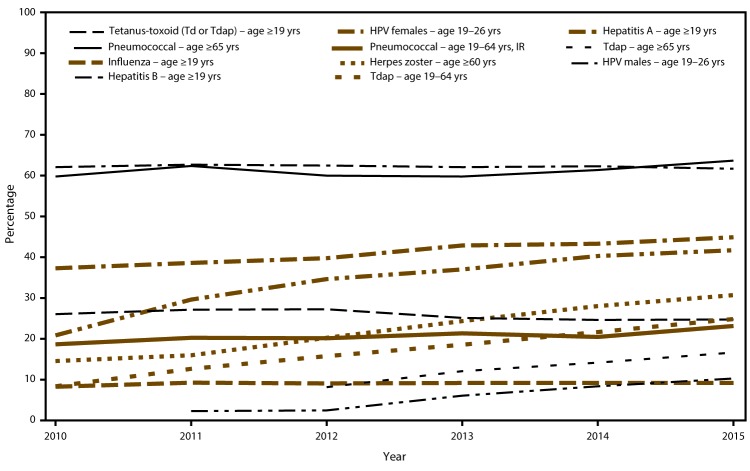
Estimated proportion of adults aged ≥19 years who received selected vaccines,* by age group and increased risk status^^†^^ — National Health Interview Survey, United States, 2010–2015 **NOTE:** Additional tables for this figure are available at https://stacks.cdc.gov/view/cdc/44991. **Abbreviations:** HPV = human papillomavirus; IR = increased risk; NHIS = National Health Interview Survey; Td = tetanus-diphtheria toxoid; Tdap = tetanus, diphtheria, and acellular pertussis vaccine. * Influenza vaccination coverage for 2010 is coverage from the 2009–10 season, 2011 is coverage from the 2010–11 season, 2012 is coverage from the 2011–12 season, 2013 is coverage from the 2012–13 season, 2014 is coverage from the 2013–14 season, and 2015 is coverage from the 2014–15 season. Interviews from August through June of each season were used to estimate coverage from July through May using Kaplan Meier survival analysis. Tdap vaccination coverage data among adults aged ≥65 years are available beginning in the NHIS 2012 survey. The 2010 HPV vaccination coverage estimate among males is suppressed due to relative standard error >30%. ^^†^^ Adults were considered at increased risk for pneumococcal disease if they had ever been told by a doctor or other health professional that they had diabetes, emphysema, chronic obstructive pulmonary disease (beginning in 2012), coronary heart disease, angina, heart attack, or other heart condition; had a diagnosis of cancer during the previous 12 months (excluding nonmelanoma skin cancer); had ever been told by a doctor or other health professional that they had lymphoma, leukemia, or blood cancer; had been told by a doctor or other health professional that they had chronic bronchitis or weak or failing kidneys during the preceding 12 months; had an asthma episode or attack during the preceding 12 months; or they were current smokers.

### Influenza Vaccination

Overall, less than 45% of adults aged ≥19 years were vaccinated annually during the influenza seasons spanning the 2009–10 through 2014–15 seasons, well below the *Healthy People 2020* target of 70% for annual vaccination of adults against seasonal influenza. Vaccination coverage among HCP with and without direct patient care also remained far below the *Healthy People 2020* target for HCP of 90%. Previous studies of influenza illnesses and hospitalizations that could be averted by vaccination have indicated that higher vaccination rates could have resulted in prevention of a substantial number of influenza cases and hospitalizations (*11*,*12*). More effort is needed to reach the *Healthy People 2020* targets to benefit more fully from influenza vaccines. Ensuring that all persons who visit a health care provider during the influenza season receive a vaccination recommendation and offer from their provider and use of vaccination information systems could increase influenza vaccination rates (*13*,*14*). Implementing interventions shown effective in increasing uptake of influenza vaccination among HCP including access to vaccination at the workplace at no cost for >1 day could improve coverage in this population (*15*–*17*).

### Pneumococcal Vaccination

The proportion of pneumococcal vaccination by type of vaccine (PCV13 or PPSV23) was not measured in the 2015 NHIS. The overall pneumococcal vaccination estimates in this report include respondents who received PCV13 and/or PPSV23. Ascertaining type-specific pneumococcal vaccination via self-report through survey questions presents challenges, particularly because current information indicates that physicians do not routinely advise their patients about the types of vaccines they use (*5*) and the complexity of the pneumococcal vaccination recommendations (different for adults with no previous pneumococcal vaccination, those who might have received PCV13 previously, and PCV13-naïve adults previously vaccinated with PPSV23) (*18*–*20*). Pneumococcal vaccination of persons aged 19–64 years at increased risk and vaccination of persons aged ≥65 years increased during the 6 years covered in this report; however, both remain well below *Healthy People 2020* targets of 60% for persons aged 18–64 years at increased risk and 90% for adults aged ≥65 years. Among persons aged ≥65 years, using PCV13 in series with PPSV23 could prevent an estimated 230 cases of invasive pneumococcal disease and approximately 12,000 cases of community-acquired pneumonia over the lifetime of a single cohort of persons currently aged 65 years through life expectancy (*18*). Achieving higher pneumococcal vaccination levels could improve these benefits.

### Tetanus Toxoid-Containing Vaccination

A single dose of Tdap is recommended for all adults aged ≥19 years who have not yet received a dose, including those aged ≥65 years, and should be administered regardless of interval since the most recent Td (*21*). Although there were modest increases in Tdap vaccination of adults from 2010 to 2015, coverage has remained low for all age groups and among adults living with an infant aged <1 year. Recent studies have shown that Tdap vaccination is highly effective at preventing pertussis among adolescents in the short-term; however, there is rapid waning of immunity in the years following Tdap receipt (*22*–*28*). Evidence indicates that the duration of protection wanes more quickly from acellular pertussis-containing vaccines than immunity derived from whole-cell pertussis vaccine formulations, and that the type of priming dose received (i.e., acellular pertussis vaccine or whole-cell pertussis vaccine) influences the durability of immunity (*22*–*24*,*29*,*30*). There is evidence that receiving at least a single dose of whole-cell pertussis vaccine, especially as the first dose of a pertussis-containing vaccine series, provides greater and longer-lived protection, irrespective of the type of subsequent doses (*22*–*24*,*29*,*30*). In the United States, booster doses with acellular pertussis-containing vaccines for infants and children (the fourth and fifth doses of the childhood series administered at ages 15–18 months and 4–6 years, respectively) were first recommended to replace whole-cell pertussis vaccine formulations in 1992 and primary doses administered at ages 2, 4, and 6 months were recommended in 1997 (*22*–*24*). Given the timing of the transition, many young and older adults would have been primed with whole-cell pertussis vaccine and might have more durable immunity (*22*,*24*,*26*). Despite the observed limitations of Tdap, current vaccination strategies remain the best approach to reducing the burden of pertussis among adolescents and adults (*24*). Health care providers should not miss an opportunity to vaccinate adults aged ≥19 years who have not received Tdap previously. Pregnant women are recommended to receive a dose of Tdap during every pregnancy, optimally between 27 and 36 weeks gestation, to provide protection to young infants through maternal antibody transfer (*31*).

### Hepatitis A Vaccination

Hepatitis A vaccination is recommended for persons traveling to or working in countries that have high or intermediate endemicity of hepatitis A, if some risk factor is present (e.g., on the basis of lifestyle, occupation, or medical condition) or for any person seeking protection from hepatitis A virus infection (*6*). Information was available only for those with foreign travel to areas of high or intermediate endemicity and those with chronic liver disease. Although hepatitis A vaccination of adults who had traveled outside the United States to a country in which hepatitis A is of high or intermediate endemicity was higher in 2015 and preceding years than among adults who did not travel outside the United States or had traveled only to countries in which the disease is of low endemicity, overall hepatitis A vaccination among travelers and persons with chronic liver disease has remained low. Health care providers are encouraged to assess the needs of their patients for hepatitis A vaccine and offer it whenever appropriate.

### Hepatitis B Vaccination

Hepatitis B vaccination coverage in 2015 among persons with diabetes showed no improvement over estimates obtained before this recommendation, which underscores the need to improve awareness of increased risk for contracting acute hepatitis B among persons with diabetes and to increase hepatitis B vaccination in this population. Similar to hepatitis A vaccination, overall hepatitis B vaccination among travelers and persons with chronic liver disease has remained low, although hepatitis B vaccination of persons who had traveled outside the United States to a country in which hepatitis B is of high or intermediate endemicity was higher in 2015 and preceding years than among respondents who did not travel outside the United States or had traveled only to countries in which the disease is of low endemicity. During 2010–2015, estimates of hepatitis B vaccination among HCP have not improved, ranging from 61%–65%, well below the *Healthy People 2020* target of 90%.

### Herpes Zoster Vaccination

Herpes zoster vaccination coverage for adults aged ≥60 years was 30.6% in 2015, a 2.7 percentage point increase compared with 2014, and 0.6 percentage points above the *Healthy People 2020* target of 30%. Although the *Healthy People 2020* target was achieved, approximately 70% of adults recommended to receive this vaccine remain unprotected. Although shortages of herpes zoster vaccine that likely contributed to low uptake during the first years after licensure were resolved, other barriers persist, particularly the high cost for providers to purchase a supply, challenges to stocking the vaccine (which requires freezer storage), coverage for the vaccine under Medicare Part D, which results in billing challenges for medical providers other than pharmacist vaccine providers, and out-of-pocket payments for some Medicare Part D beneficiaries depending on their specific plan (*32*,*33*). Although health care provider recommendations for vaccination are strongly associated with a patient’s receipt of vaccines (*9*,*13*,*14*), individual awareness of vaccine-preventable disease consequences can also influence vaccination behavior. For example, rates of herpes zoster vaccination were increased among persons who witnessed friends or family members experience herpes zoster (*34*), particularly if the herpes zoster was severe (CDC, Division of Viral Diseases, National Center for Immunization and Respiratory Diseases, unpublished data, 2016). In one study (*34*), blacks had a lower rate of self-reported prevalence of herpes zoster and of witnessing friends or family members with herpes zoster compared with whites. These factors might have influenced perceived risk for herpes zoster among blacks in that study, their interest in herpes zoster vaccination, and contributed to the lower vaccination levels among blacks observed in this study population (*34*). Vaccination with the current live-attenuated herpes zoster vaccine is contraindicated in immunocompromised persons (*35*). Studies and clinical trials of other herpes zoster vaccines have been conducted to identify immunizing agents that could protect more population groups, including selected populations of immunocompromised patients considered at increased risk for herpes zoster infection and complications (*36*–*42*) and older adults (*40*–*42*).

### HPV Vaccination

ACIP has recommended routine vaccination at age 11 or 12 years for girls since 2006 and for boys since 2011. ACIP also recommends vaccination for females aged 13–26 years and for males aged 13–21 years who have not been vaccinated previously or who have not completed the 3-dose series; males aged 22–26 years may be vaccinated (*43*,*44*). Although vaccination coverage has increased since a licensed HPV vaccine has been available and recommended by ACIP, many adolescent and young adult females and males remain unvaccinated and vulnerable to develop cancers that safe, effective HPV vaccines can prevent (*5*,*43*,*45*).

In 2015 among women and men aged 19–26 years, 6.2% and 6.9% reported receiving the first dose of HPV vaccine at age 11–12 years, respectively, with most (91.8% of females and 88.8% of males) reporting receipt at age ≥13 years, consistent with the fact that female respondents aged ≥22 years and all male respondents would have been aged >13 years at the time HPV vaccination was first recommended. In 2015, approximately 12% of females and 3% of males aged 19–26 years not vaccinated at age ≤18 years reported receiving the first dose of HPV vaccine as catch-up at age 19–26 years. Since HPV vaccine licensure, multiple cohorts of unvaccinated adolescents and young adults have accumulated. Based on 2015 data alone, as many as 9.1 million women and approximately 13.9 million men aged 19–26 years were unvaccinated and might benefit from HPV vaccination assuming no contraindications to vaccination. Until HPV vaccination increases among adolescents, a high proportion of unprotected young women and men eligible for HPV vaccination will be expected. For example, in the 2015 National Immunization Survey – Teen (*45*), provider-reported vaccination histories indicated that among females and males aged 17 years, 29.4% (582,947) and 49.6% (980,637), respectively, were unvaccinated (having not received at least one HPV vaccine dose) (*45*). These estimates reflect the current pool of females and males who could benefit from catch-up vaccination and the number of unprotected older adolescents adding to that pool annually. Studies have found that although HPV infection increases with increasing age after sexual debut, most women have not been infected with all the high risk HPV types targeted by the vaccines (*46*,*47*), supporting implementation of ACIP-recommended catch-up vaccination, because vaccination can protect against HPV types for which vaccination candidates have not been infected. Results from modeling and studies of the cost-effectiveness of HPV vaccination of young women and men suggest that catch-up vaccination could reduce the amount of time needed to achieve population level impacts of vaccination on infections with HPV vaccine types and sequelae such as cancer (*48*–*55*). Findings from initial studies of vaccine impact in settings in which catch-up vaccination programs were successful in achieving high coverage rates among young women are consistent with these models (*56*–*59*). Although cost-effectiveness studies indicate that catch-up vaccination might become less favorable over time, a long-term strategy of HPV vaccination of young adult females through age 18–26 years could be considered cost-effective and, when combined with an adolescent vaccination program, appears to represent an effective strategy (*49*,*50*,*54*,*55*,*60*–*67*). Data for the years 2008–2012 from population-based cancer registries (that participate in CDC’s National Program of Cancer Registries and the National Cancer Institute’s Surveillance, Epidemiology, and End Results program) indicated that of the average of 38,793 HPV-associated cancers diagnosed annually (11.7 per 100,000 persons; including 23,000 [13.5] among females and 15,793 [9.7] among males), 30,700 (79%) were estimated to be attributable to HPV based on polymerase chain reaction genotyping studies (*68*). Among these HPV-associated cancers, 24,600 (80%) were attributable to HPV types 16 and 18, which can be prevented by HPV vaccines (bivalent, quadrivalent and 9-valent vaccines), and 3,800 (12%) were attributable to the five additional HPV types which can be prevented by the 9-valent vaccine (HPV types 31, 33, 45, 52, 58). Although these findings represented an overall increase in HPV-associated cancer incidence from 10.8 per 100,000 persons to 11.7 per 100,000 persons, compared with a previous analysis which reported cases diagnosed annually during 2004–2008 (*69*), impact of vaccination activities on HPV-attributable cancers will not be observed for decades after vaccine introduction. However, impact of HPV vaccination programs already has been observed on HPV prevalence, genital warts and cervical precancers (*70*–*78*). Increasing HPV vaccination coverage could lead to greater decreases in HPV attributable diseases in the United States. Continued efforts are needed to improve coverage among members of the primary target group for HPV vaccine, girls and boys aged 11–12 years (*79*), and among all racial and ethnic groups. To reduce the amount of time needed to achieve population level impacts of vaccination such as reduction in HPV-associated cancer incidence, efforts also are needed to improve catch-up vaccination among those who have not started or completed their vaccination.

### Racial and Ethnic Differences in Vaccination

Compared with 2014, racial/ethnic differences in vaccination coverage persisted for all seven vaccines in this report. Generally higher coverage was observed for whites compared with most other groups. These differences widened for pneumococcal and herpes zoster vaccination (due primarily to increases among whites). Blacks, Hispanics, and Asians had lower vaccination coverage than that of whites for all of the vaccines routinely recommended for adults, with just a few exceptions. Among HCP, there were differences for influenza, Tdap, and hepatitis B vaccination, with white HCP generally having higher vaccination coverage compared with black and Hispanic HCP.

The findings provided in this report are consistent with those from a study on racial and ethnic disparities in vaccination coverage among adults using 2012 NHIS data (*80*). Five vaccines were included in this study (influenza, pneumococcal [both PPSV23 and PCV13], tetanus (Td), herpes zoster, and HPV). There were vaccination disparities for most other groups compared with whites for 17 of the 24 comparisons by vaccine and age/target groups, even after adjusting for demographic and access-to-care characteristics. In most of the logistic models, whites reported receipt of vaccinations more often than blacks, Hispanics, and Asians after controlling for other demographic and access-to-care characteristics. Factors that were independently associated with receipt of most of the examined vaccines included race and ethnicity, age, sex, education, health insurance, and having a usual place for health care. The number of physician visits in the past 12 months was independently associated with receipt of all the vaccinations assessed in this study. Results from this study indicated that racial and ethnic differences in vaccination levels narrow when adjusting for socioeconomic factors analyzed in this survey, but are not eliminated, suggesting that other factors that are associated with vaccination disparities are not measured by the NHIS and could also contribute to the differences in coverage.

Previous research has indicated a variety of factors that contribute to racial/ethnic differences in adult vaccination rates, including patient, provider, and system factors (*81*–*84*). Standardized offering of vaccines reduces these differences (*85*,*86*). Using a combination of patient tracking, vaccination reminders for providers and patients, and patient outreach and assistance also reduces racial/ethnic vaccination differences (*87*). Incorporating standards for adult vaccination practices, which include routinely assessing vaccination needs during clinical encounters, providing a strong recommendation for vaccination to patients with indications, and then offering vaccination at the visit (*14*), can reduce vaccination disparities.

### Improving Adult Vaccination Coverage

Adult vaccination coverage remains low for most routinely recommended vaccines. Racial/ethnic differences in coverage persisted for all seven vaccines in this report with higher coverage, generally, for whites compared with most other groups. Factors that contribute to low adult vaccination rates (including patient- and provider-level barriers and systems-related factors) have been described (*5*,*88*–*96*) and effective, evidence-based strategies to improve vaccine use have been reported (*13*,*88*). The National Vaccine Advisory Committee published updated standards for adult immunization practice in 2014 with the intent of improving adult vaccination coverage of ACIP-recommended vaccines. This guidance calls on health care providers, including those who do not stock vaccines, to 1) assess the vaccination status of patients at every clinical encounter; 2) recommend needed vaccines for patients; 3) offer recommended vaccines or, for providers who do not stock a needed vaccine, refer patients to a vaccine provider; and 4) document vaccines administered, including in immunization information systems when available for use among adult patients (*14*). Nationwide adoption of electronic health records (EHRs), electronic patient portals, and patient-directed clinical decision support delivered via patient portals offer opportunities for innovative approaches to improving adult vaccination rates. For example, to improve influenza vaccination among eligible adult patients, a protocol is being evaluated in a large multispecialty group practice. The protocol uses patient-directed clinical decision support involving EHR patient portal messages and interactive voice recognition calls to promote influenza vaccination and obtain patient self-report of vaccines received outside the practice as well as information on barriers to vaccination (*97*). To improve administration and documentation of receipt of Tdap, consensus-based teams of clinicians, nurses, medical assistants, and other support staff have used an automated clinical reminder system to provide patient-specific vaccination recommendations to clinicians at the point of care (*98*). Seven large medical groups participating in an adult immunization best practices collaborative in six states have used a clinical data repository comprised of claims data, billing data, and data from electronic clinical systems to increase influenza vaccination in adults aged ≥18 years and pneumococcal vaccination successfully among groups at increased risk for invasive pneumococcal disease (*99*,*100*). Various strategies have been evaluated for increasing influenza, Tdap, and HPV vaccination in select populations such as pregnant women and women in the general population, including interventions to overcome provider and system barriers (e.g., physician education and reminders) (*101*), interventions to increase demand for vaccination (e.g., text messaging encouraging vaccination in an ambulatory obstetric population) (*102*), and interventions to enhance vaccine access (*103*–*107*). The expanded availability of vaccine services in pharmacies and other retail settings has also improved vaccine accessibility (*108*–*110*). To increase targeted vaccination coverage, the Task Force on Community Preventive Services recommends a combination of strategies that include selected interventions from two or three categories: increasing community demand for vaccinations, enhancing access to vaccination services, and provider- or system-based interventions (*13*,*111*).

## Limitations

The findings in this report are subject to at least eight limitations. First, the NHIS sample excludes persons in the military and those residing in institutions, which might result in underestimation or overestimation of vaccination coverage levels. Second, the response rate was 55.2%. Nonresponse bias can result if respondents and nonrespondents differ in their vaccination rates. Third, the determination of vaccination status and identification of high-risk conditions in NHIS were not validated by medical records. Fourth, self-report of vaccination might be subject to recall bias. Young adults (e.g., persons aged 19–26 years), particularly, might not be able to recall accurately vaccines received as infants or adolescents. However, adult self-reported vaccination status has been shown to be ≥70% sensitive in one or more studies for influenza, pneumococcal, tetanus toxoid-containing, herpes zoster, and hepatitis B vaccines and ≥70% specific in one or more studies for all except tetanus and hepatitis B vaccination (*112*–*115*). Fifth, demographic and other characteristics (e.g., insurance status, usual source, and frequency of health care) were self-reported and were not validated. Sixth, the Tdap estimate is subject to considerable uncertainty. Respondents who reported a tetanus vaccination but were unable to say whether Td or Tdap was used during 2005–2015 were excluded from estimations of Tdap coverage, creating a potential for bias. Sensitivity calculations were conducted to assess the magnitude of potential bias. Depending on what proportion of excluded respondents actually received Tdap, Tdap coverage could fall within the range of 15.3%–49.3% for adults aged ≥19 years, 16.3%–50.4% for adults aged 19–64 years, and 11.0%–44.5% for adults aged ≥65 years. To assess the potential impact of excluding respondents from estimation of Tdap coverage, multiple imputation was used to impute missing Tdap vaccination status among respondents who reported a tetanus vaccination but were unable to say whether Td or Tdap was received and these estimates were compared with Tdap estimates derived by excluding these respondents. The multiple imputation model included age, gender, race/ethnicity, marital status, education, employment status, poverty level, number of physician contacts in the past year, usual source of care, self-reported health status, nativity, and four regions of U.S. residence defined in the NHIS (Northeast, Midwest, South, and West). The estimates derived by excluding respondents who were unable to say whether Td or Tdap was received and by multiple imputation differed by less than one percentage point for respondents aged ≥19 years, aged 19–64 years, and those aged ≥65 years (differences: 0.6, 0.7, and 0.3, respectively). Comparisons of Tdap coverage across years within subgroups also might be affected by bias resulting from excluding persons who did not report the type of tetanus vaccine they received. Seventh, age at vaccination is not known for vaccines adults reported having “ever” received (e.g., pneumococcal, hepatitis B, HPV and herpes zoster vaccines), so it is not clear for younger adults whether vaccination occurred as an adult or as part of a child or adolescent vaccination program (hepatitis B and HPV vaccination) or among older adults how to interpret age-specific findings. These data might provide a reasonable measure of population protection but not of year to year programmatic change. Finally, the prevalence of selected behavioral characteristics in populations, including the use of preventive health services, vaccine safety concerns, state laws and vaccination intervention programs, cultural, religious, and other factors might affect vaccination coverage. Although NHIS collects information on use of preventive health services other than vaccination, this information was not included in this analysis. NHIS did not collect information on the other factors listed above.

## Conclusions

The findings provided in this report indicate that vaccination coverage levels among U.S. adults are not optimal. Improvement in adult vaccination is needed to reduce the health consequences of vaccine-preventable diseases among adults. Awareness of the need for vaccines for adults is low among the general population, and adult patients rely on provider recommendations for vaccination (*88*–*91*,*93*). Successful vaccination programs combine 1) education of potential vaccine recipients and publicity to promote vaccination; 2) increased access to vaccination services in health care settings; and 3) use of practices that improve vaccination coverage, including reminder-recall systems, efforts to remove administrative and financial barriers to vaccination, use of standing order programs for vaccination, and assessment of practice-level vaccination rates with feedback to staff members (*13*,*14*,*116*). Health care provider recommendations for vaccination are strongly associated with a patient’s receipt of vaccines (*87*,*117*–*119*). Incorporating routine assessment of adult patient vaccination needs, recommendation, and offer of needed vaccinations for adults into routine clinical care of adults can help improve vaccination rates and narrow widening racial and ethnic disparities in vaccination coverage (*13*,*14*). The adult immunization schedule (*6*), updated annually, provides current recommendations for vaccinating adults and a ready resource for persons who provide health care services for adults in various settings. Assessing the associations between vaccination and sociodemographic and other factors is important for understanding factors that contribute to low coverage rates and to disparities in vaccination, and for implementing strategies to improve vaccination coverage.

## Footnotes

* In 2006, a single dose of Tdap was recommended for adults aged 19–64 years, especially those who have or who anticipate having close contact with an infant aged <1 year (e.g., parents, grandparents, child care providers, and HCP) to reduce the risk for transmitting pertussis.

## Appendix

### Ascertainment of Vaccination Status

**Influenza vaccination:** Respondents were asked if they had received an influenza shot or nasal spray in the past 12 months and if so, in which month and year.

**Pneumococcal vaccination:** Respondents were asked if they had ever had a pneumonia shot. There were no questions in the 2015 NHIS to ascertain pneumococcal vaccination by type of vaccine (23-valent pneumococcal polysaccharide vaccine or 13-valent pneumococcal conjugate vaccine).

**Tetanus vaccination:** Respondents were asked if they had received a tetanus shot in the past 10 years. Respondents who had received a tetanus shot in the past 10 years were asked if their most recent shot was received in 2005 or later. Respondents who had received a tetanus shot since 2005 were asked if they were told that their most recent tetanus shot included the pertussis or whooping cough vaccine.

**Hepatitis A vaccination:** Respondents were asked if they had ever received the hepatitis A vaccine, and if yes, how many doses were received.

**Hepatitis B vaccination:** Respondents were asked if they had ever received the hepatitis B vaccine, and if yes, whether they had received ≥3 doses or <3 doses.

**Herpes zoster vaccination:** Respondents were asked if they had ever received a shingles vaccine.

**HPV vaccination:** Respondents were asked if they had ever received an HPV shot or cervical cancer vaccine, and if yes, age at the first dose.

### Classification as Health Care Personnel

Adults were classified as health care personnel if they reported that they currently volunteer or work in a hospital, medical clinic, doctor’s office, dentist’s office, nursing home, or some other health care facility including part-time and unpaid work in a health care facility as well as professional nursing care provided in the home.

### Ascertainment of Health Insurance Status

Adults were considered insured if they reported having public health insurance coverage (Medicare, Medicaid, military health care [TRICARE/VA/CHAMP-VA], Indian Health Service, state-sponsored health plan, or other government program insurance) or private health insurance coverage.

### Ascertainment of Having a Usual Place for Health Care

Respondents were asked if there is a place to which they usually go when sick or need advice on their health. Respondents answering “yes” were defined as having a usual place for health care.
